# Ethnobotanical study on factors influencing plant composition and traditional knowledge in homegardens of Laifeng Tujia ethnic communities, the hinterland of the Wuling mountain area, central China

**DOI:** 10.1186/s13002-024-00742-4

**Published:** 2024-12-02

**Authors:** Shuwang Hou, Mengfan Yu, Zhen Yao

**Affiliations:** College of Horticulture and Gardening, Yangtze University, Jingzhou, 434025 China

**Keywords:** Homegarden, Ethnobotany, Local knowledge, Tujia ethnic group, Wuling mountain area

## Abstract

**Background:**

A homegarden is a conventional small-scale agricultural ecosystem dominated predominantly by humans. Homegarden plants, which are plants with specific functionalities that are either cultivated or consciously preserved within the homegarden, are the most critical elements of the homegarden and are capable of providing a multitude of products and services. Recognized as one of China’s biodiversity hotspots, the Wuling mountain area has long been inhabited by multiple ethnic groups, each of which has accumulated a wealth of traditional knowledge. This study focuses on the Tujia ethnic communities in Laifeng County, situated in the hinterland of the Wuling mountain area, with the primary objective of collecting, documenting, and organizing local homegarden plants, their functions, and the associated traditional knowledge, and exploring the factors influencing their composition and diversity.

**Methods:**

From May 2023 to August 2024, field surveys were conducted in Laifeng County, Enshi Tujia and Miao Autonomous Prefecture, Hubei Province, China. Semi-structured interviews and participatory observation were used to gather information on homegarden plants with informed consent. To analyze and evaluate homegarden plants, we employed the relative frequency of citation (*RFC*) and Jaccard index (*J*) for quantitative analysis. Additionally, the factors influencing the composition of homegarden plants were investigated using redundancy analysis (RDA).

**Results:**

This study documented a total of 414 species of homegarden plants from 114 families. These plants are primarily sourced from market purchases (158), spontaneous species (107), wild introductions (103), neighborhood sharing (59), and self-preservation (36). Homegarden plants serve multiple functions, including ornamental (201), medicinal (189), edible (165), traded (95), timber (34), forage (28), and other functions (11). There are 24 homegarden plants with an *RFC* greater than 0.5, indicating their high importance, including *Allium fistulosum*, *Raphanus sativus*, and *Brassica rapa* var. *chinensis*. The Jaccard index results suggest the highest degree of similarity between the homegarden plants in Geleche Town and Dahe Town. The RDA results established that knowledge of herbal medicine and homegarden area are two significant factors impacting the composition and diversity of homegarden plants, with the average age of the household resident population also exerting an influence.

**Conclusions:**

This study reveals the composition, source diversity, and functional diversity of homegarden plants within the Laifeng Tujia ethnic community, along with the influencing factors. These homegarden plants play an integral role in sustaining the balance of the homegarden ecosystem and supporting the daily lives of local residents. The management of homegarden plants by the Laifeng Tujia ethnic community carries traditional agricultural knowledge and wisdom. Emphasis should be placed on bolstering the understanding, protection, and transmission of traditional knowledge and culture related to homegardens, which play a vital role in safeguarding local agricultural biodiversity and fostering sustainable development.

**Supplementary Information:**

The online version contains supplementary material available at 10.1186/s13002-024-00742-4.

## Introduction

A homegarden represents a conventional small-scale traditional agricultural ecosystem predominantly managed by humans, and is typically positioned as centralized management land near homesteads [1, 2]. They are generally delineated by elements such as bamboo, shrubs, or other materials and are utilized for the cultivation of vegetables, fruits, and ornamental plants, as well as for animal production [3, 4]. The homegarden serves diverse functions and roles, including food production, medicinal applications, ornamental purposes, material provisions, and cultural significance [5–7]. Furthermore, the homegarden features a high degree of species diversity and plant genetic diversity, rendering it an invaluable repository of local biodiversity and making substantial contributions to the protection of rare, endangered, or overexploited species [8–10]. Additionally, the products of homegardens often constitute a substantial source of supplementary cash income for numerous families [6, 11].

Homegarden plants, cultivated or consciously conserved in the homegarden, perform specific functions. They serve as the most critical component of the homegarden, catering to various product and service requirements of daily family life [9]. Previous studies have categorized homegarden plants into groups on the basis of their respective functions and uses. The homegarden plants used for food primarily serve the purpose of self-sufficiency. For instance, in the homegarden woody plants of northern Thailand, the majority are edible fruit trees, with *Mangifera indica* being the most prevalent [12]. In addition to providing food for family members, several types of homegarden plants also serve as forage for poultry and livestock [13]. Homegardens are also recognized globally as important habitats for medicinal plants, preserving a large amount of traditional knowledge [14, 15]. Among the homegardens of four ethnic groups in Thailand (Thai Yuan, Karen, Lahu, and Lisu), 95 types of medicinal plants have been identified, which are mainly used for treating infection and infestation, nutritional disorders, and digestive system disorders [6]. Moreover, ornamental plants constitute a significant component of homegarden plants [16]. For instance, ornamental plants are used extensively in the homegardens of the Baiku Yao settlement in southern China, for decoration and to admire while relaxing, particularly in relocated villages [16]. In addition to being self-sufficient, certain homegarden plants are also utilized for commercial purposes [17].

The composition of homegarden plants is influenced by various factors, including differences in household characteristics and natural conditions. Among the factors, household landholdings, income, homestead size, and time spent significantly predict the composition of homegarden plants, particularly in tropical and subtropical regions [18]. Furthermore, economic and demographic factors of households have been shown to influence the composition of homegarden plants [19–21]. These factors frequently exhibit a combined effect, collectively influencing the composition and diversity of homegarden plants. For example, in Benin, the homegarden plant diversity increased with increasing owner age and size of the homegarden [22]. In Kampung Masjid Ijok Perak, Malaysia, the size of homegardens and the income status of the homegardens owner are the most important physical and socio-economic factors that affect the diversity and composition of tree and shrub species in the homegarden [23]. Additionally, regarding the differences in natural conditions, studies have shown that communities with similar forest resource compositions may have similar homegarden plant compositions [24]. Therefore, multiple aspects should be considered when exploring the affecting factors of plant composition in homegardens.

China is a multi-ethnic country, possessing rich biodiversity and a profound historical background [25]. The history of gardening in China can be traced back to the era of the Zhou Dynasty’s “Yuanpu” (1046–256 BC) [26]. Diverse communities from various regions and ethnic groups have upheld the tradition of maintaining intact homegardens and knowledge related to the use of homegarden plants throughout their prolonged settlement process. Previous studies have predominantly focused on the southwestern regions of China (including Naxi, Baiku Yao, Tsang-la, Dai) [11, 16, 27, 28] and the Qinghai Tibet Plateau (specifically the Salar) [29].

The Wuling mountain area is located at the border of Hunan, Hubei, Sichuan, and Guizhou Provinces; has a vast area; and is the core area of China’s subtropical forest system. The area’s complex and diverse natural geographic environment, coupled with superior climatic conditions, provides a conducive environment for the formation and preservation of biodiversity, thereby marking it as biodiversity hotspot in China [30, 31]. The history of Wuling Mountain area can be traced back to the early Western Han Dynasty (around 200 BC). For more than two thousand years, diverse ethnic minorities, including Tujia, Miao, Dong, Yao and others, have established communities in this region. The mountainous terrain, dense forests, and limited access to transportation have led these ethnic minorities to adapt to the environment over time, resulting in well-preserved homegardens and associated traditional knowledge. Previous ethnobotanical studies conducted in the Wuling mountain area have substantiated this assertion to a degree. Research undertaken at Gaowangjie National Nature Reserve in Guzhang County, western Hunan Province, examined homegarden ornamental plants and put forward recommendations for their beautification [32]. Additionally, a comparative analysis was performed on the diversity of homegarden plants across three Miao communities within the Laershan region of western Hunan Province, underscoring the influence of human activities on homegarden plant diversity [24]. However, the research samples pertaining to homegarden plants from additional locations and ethnic groups within the Wuling mountain area remain limited. Furthermore, the factors influencing the composition and diversity of homegarden plants within the Wuling mountain area are yet to be clearly established, resulting in persistent and substantial research gaps.

With the enhancement of living standards and the evolution of urban homegardens, particularly in the post-COVID-19 era, homegardening has garnered considerable attention [33]. Nonetheless, the destruction of the natural environment, the expansion of urbanization, and the depopulation of rural areas have placed traditional knowledge concerning homegarden plants at risk of being lost or assimilated [29]. A comprehensive study should be undertaken to document and systematize traditional knowledge associated with homegarden plants. Moreover, factors influencing the composition and diversity of homegarden plants should be assessed to guarantee the protection and sustainable utilization of traditional knowledge of homegarden plants, thereby promoting local biodiversity conservation and regional economic growth.

This study, therefore, focuses on the Tujia ethnic communities in Laifeng County, Enshi Tujia and Miao Autonomous Prefecture, Hubei Province, located in the hinterland of the Wuling mountain area, to explore their management of homegarden plants. This study conducted an ethnobotanical survey of homegarden plants among the Laifeng Tujia ethnic community, aiming to answer the following scientific inquiries: (1) What constitutes the composition of homegarden plants within local communities? (2) What are the sources of these plants, and what functions do they primarily serve? (3) What factors influence the composition of homegarden plants within local communities? This study seeks to obtain a comprehensive understanding of the current state of homegarden plants among the Laifeng Tujia ethnic communities in the Wuling mountain area while facilitating the conservation, administration, and transmission of local homegarden plant resources and related traditional knowledge, thereby fostering rural revitalization and sustainable advancement.

## Methods

### Study area

In the southwest corner of Hubei Province (29° 06′–29° 41′N, 109° 01′–109° 27′E), Laifeng County boasts an average annual temperature of approximately 15.9 °C, typical annual precipitation of 1400 mm, a mean altitude of 680 m, and 78% of its total land area comprising of low mountains and flat dams under 800 m, which provides favorable conditions for agriculture and living [34]. It is located at the border of Hubei, Hunan, and Chongqing Provinces and has rich ecological and cultural connotations. The County is also considered one of the birthplaces of the Tujia ethnic group, with a long history and cultural heritage.

The Tujia ethnic group is one of the main ethnic minorities living in the Wuling mountainous area, and is the most populous ethnic minority in Laifeng County, with a population of 112,700. The Tujia has a long history and does not have a written language. Their spoken language belongs to the Tibetan-Myanmar language group, Sino-Tibetan family, and is close to the Yi language branch. They mainly focus on agricultural production and cultivate mountain crops.

**Fig. 1:**
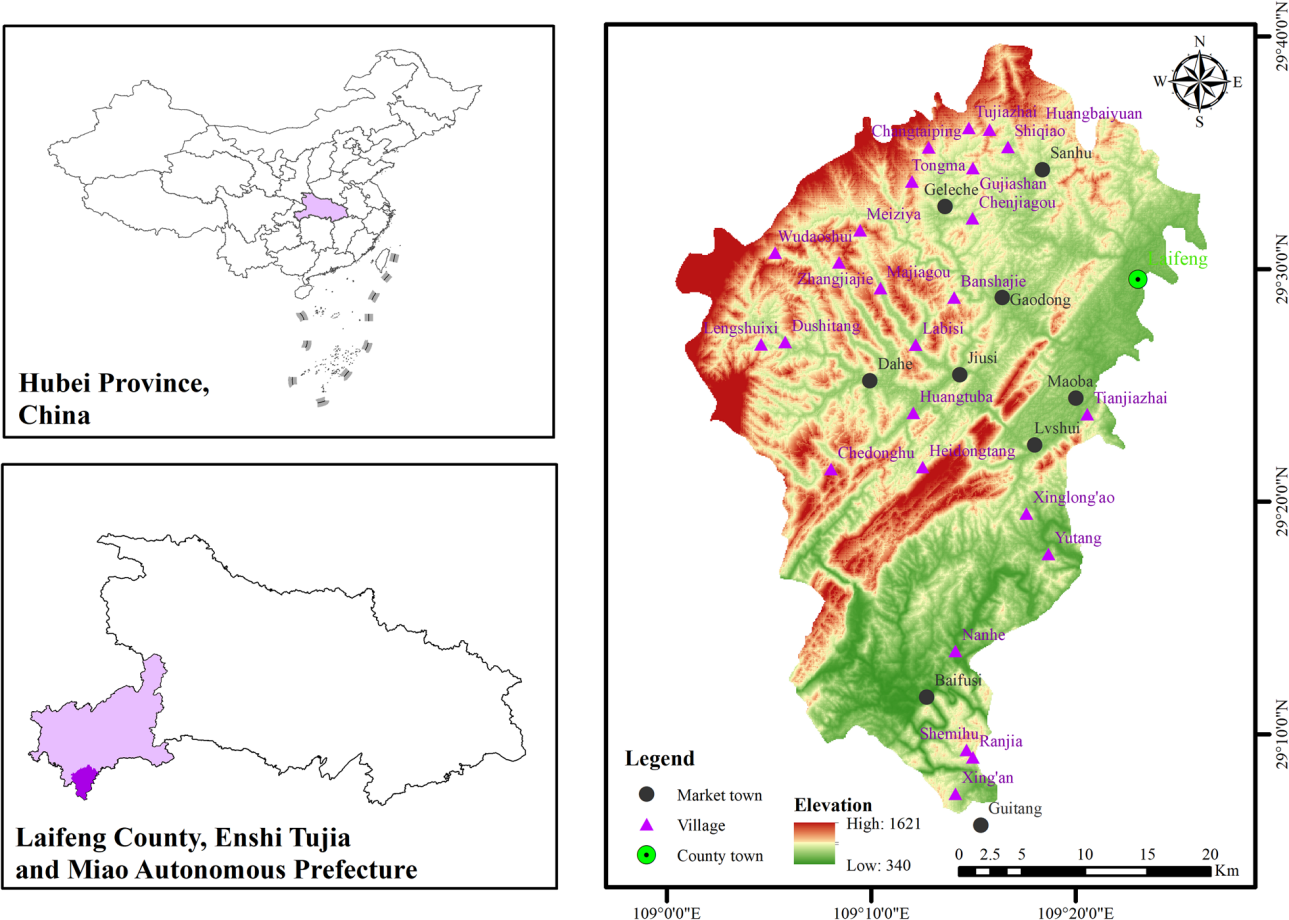
The study area. The colored map is Laifeng County, in which the surveyed villages and market towns are marked with purple triangles and black dots

According to the first to sixth batches of Chinese traditional villages, the first to third batches of Chinese ethnic minority characteristic villages, and the first to second batches of national forest villages, 25 villages in 7 towns were identified for survey (Fig. 1). The villages listed in these batches exhibit a profound history, distinct folk customs, and well preserved natural ecological features. Selecting these villages as the survey subjects can more accurately mirror the characteristics of the local communities. These villages have relatively well-preserved Tujia culture and customs and a long history of traditional mountain agriculture. Inhabitants in some villages condense within minor courtyards, giving rise to continuous clusters of stilted houses and homegarden landscapes, thereby maintaining a fairly unbroken understanding of homegardens.

### Field survey and data collection

**Fig. 2:**
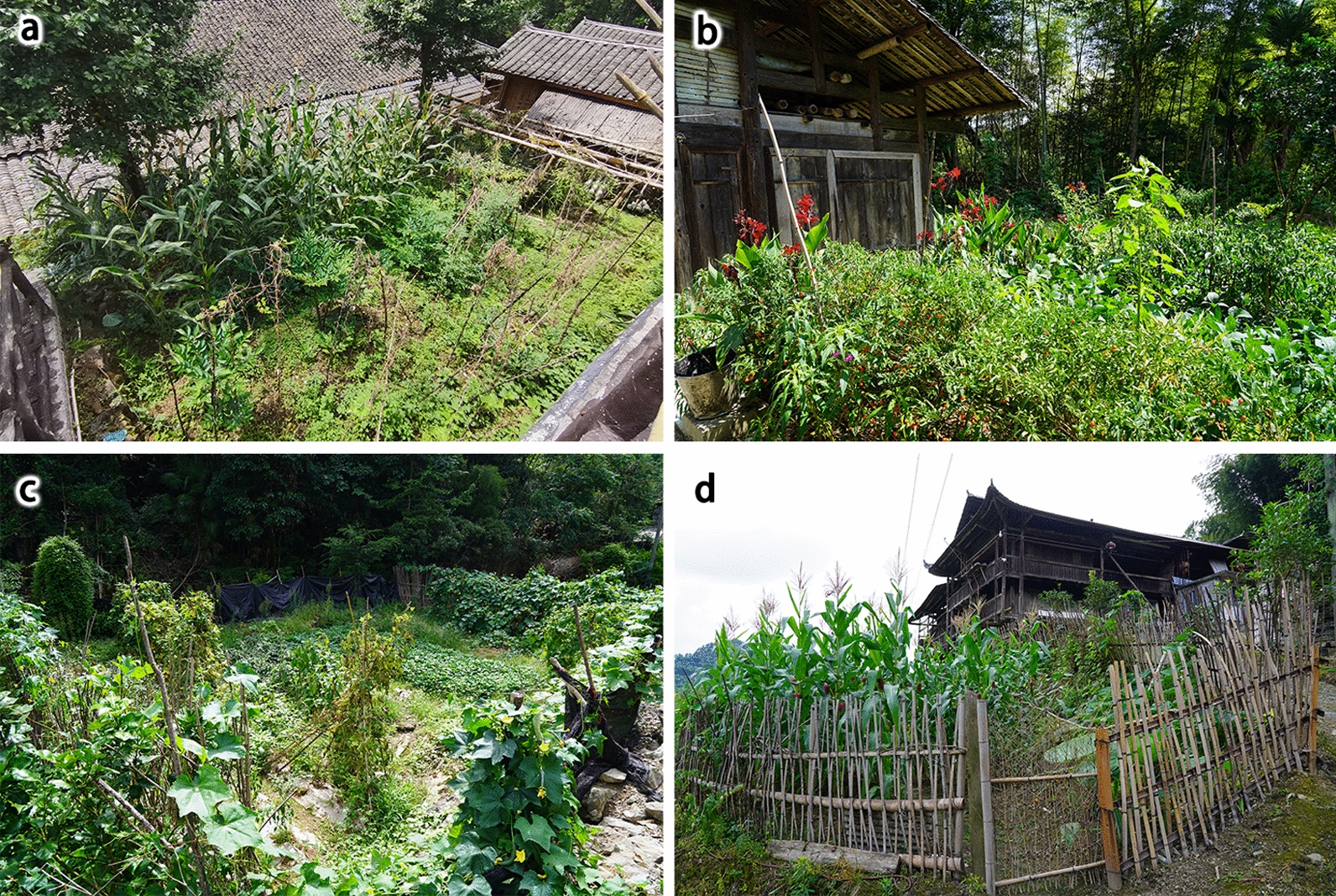
Homegarden of Laifeng Tujia ethnic communities. **a** Wudaoshui Village, Dahe Town; **b** Xing’an Village, Baifusi Town; **c** Chedonghu Village, Dahe Town; **d** Meiziya Village, Jiusi Town

The survey was conducted in different seasons from May 2023 to August 2024 within the territory of Laifeng County. During the survey, we randomly selected homegardens within the selected villages. When selecting a homegarden, we follow the following criteria: (1) Near the house; (2) defined boundaries (fences made of bamboo, bricks, and other materials, or separation by planting trees); (3) the total area should be at least 66.67 m^2^ (equivalent to 1/10 mu, “mu” is the unit of land area in China’s municipal system, 15 mu = 1 hectare); and (4) someone continuously manages and maintains it, and it has not been abandoned. To ensure random sampling and reduce bias, we selected 8–12 well-preserved homegardens with traditional management in each village (Fig. 2).

A total of 243 homegardens were surveyed, and geographical location information is recorded in the supplementary materials (Additional file 1: Table S1). Firstly, we visited the local community committee for getting field study permission. Before conducting the homegarden plant survey, we briefly reported our survey objectives to the households and obtained their verbal consent. Data collection is conducted with the verbal consent of the participants. During the survey, we conducted semi-structured interviews using local dialects to collect data [29]. The survey procedure adhered to the ethical guidelines of the International Society of Human Ethology [35]. The questions we asked mainly the following:

1. What are the main plants in your homegarden?
2. What are the main functions of these plants?
3. How do you use these plants?
4. What are the main sources of these plants?
5. Which parts of these plants are mainly used?

In addition to using semi-structured interviews to gather information, we also invited family members to introduce the plants planted in the homegarden and recorded the preserved species of homegarden plants. Additionally, we recorded relevant information on the characteristics of the homegarden, such as altitude, distance to the nearest market, distance to the county town, number of permanent residents in the household, average age of permanent residents, average years of education, homegarden area, and whether households are herbalists or have knowledge of herbals (Additional file 1: Table S1). Table 1 shows the market location and date information for different villages.

**Table 1:** The market location and market time for Laifeng Tujia ethnic communities

Market town	Location	Market time (lunar)	Village
Dahe	29° 25′ 41″ N, 109° 11′ 2″ E	2nd, 5th, 8th	Huangtuba, Wudaoshui, Chedonghu, Dushitang, Lengshuixi
Jiusi	29° 25′ 53″ N, 109° 15′ 31″ E	3rd, 6th, 9th	Heidongtang, Majiagou, Labisi, Zhangjiajie
Gaodong	29° 29′ 11″ N, 109° 17′ 28″ E	1st, 4th, 7th	Banshajie
Maoba	29° 24′ 35″ N, 109° 21′ 11″ E	2nd, 5th, 8th	Tianjiazhai
Guitang	29° 6′ 22″ N, 109° 15′ 42″ E	3rd, 8th	Ranjia, Xing’an
Baifusi	29° 12′ 0″ N, 109° 13′ 22″ E	2nd, 5th, 8th	Shemi, Nanhe
Geleche	29° 33′ 1″ N, 109° 15′ 9″ E	2nd, 5th, 8th	Chenjiagou, Tongma, Changtaiping, Gujiashan
Sanhu	29° 34′ 25″ N, 109° 19′ 55″ E	1st, 4th, 7th	Tujiazhai, Shiqiao
Lvshui	29° 22′ 38″ N, 109° 19′ 7″ E	3rd, 6th, 9th	Xinglong’ao, Yutang

Simultaneously, voucher specimens and plant photos were collected with the informed consent of the households. For native and local featured plants, we collected voucher specimens, and for common cultivated plants, we took photographs as voucher specimens and marked them in the catalog table [27]. The identification of plant species primarily relied on the books (*Flora of China*, *Flora of Hubei*, and *Flora of Enshi*) and electronic online resources (http://www.iplant.cn/, https://www.worldfloraonline, and http://www.sp2000.org.cn/). All vascular plants were named online according to World Flora Online (https://www.worldfloraonline). The voucher specimens are stored at the herbarium of the College of Horticulture and Gardening, Yangtze University, in Jingzhou City, Hubei Province.

The market time refers to the specific day designated for small vendors to set up stalls for trading, a customary practice in the town. On this designated day, residents from surrounding villages congregate in the town to carry out their buying and selling activities. The market time is calculated according to the lunar calendar. For example, in Daheji market town, markets are held on the 2nd, 5th, and 8th of each month, which is the lunar calendar’s market day on the 2nd, 5th, 8th, 12th, 15th, 18th, 22nd, 25th, and 28th of each month.

### Relative frequency of citation (RFC)

The importance of homegarden plants in the daily livelihood of Laifeng Tujia ethnic communities was evaluated via the relative frequency of citation [36]. The *RFC* value calculation formula is as follow:

*RFC* = $$\frac{{FC}_{s}}{N}$$

*FCs* represent the frequency of citation (total number of frequencies mentioned by all respondents to a specific plant), and N represents the number of all homegardens surveyed [36].

The *RFC* value is between 0 and 1. The higher the *RFC* is, the closer the connection between homegarden plants and the daily livelihood of the Tujia ethnic communities in Laifeng.

### Jaccard index (J)

The Jaccard index was used to detect the similarity of plants species between homegardens among the different towns [37]. The index was calculated via the following formula:

$$J=\frac{c}{a+b-c}$$

Where “*a*” = the number of homegarden plants owned by village in the town 1; “*b*” = the number of homegarden plants owned by village in the town 2; and “*c*” = the number of plants commonly used in the homegardens of the two towns [37]. The *J* value is between 0 and 1. The higher the *J* value is, the greater the similarity of household garden plants between two towns. The results of Jaccard values are presented using a heatmap.

The Jaccard distance (*JD*) was used to visualize the value of the Jaccard index [27].

$$JD=1-J$$

Multidimensional scaling analysis is performed on the Jaccard distance values via ALSCAL in SPSS 26, and then scatter plots are plotted via the Euclidean distance model [27]. After 4 iterations in two-dimensional solution, the improvement of S-stress value is 0.00031, which is lower than the default 0.001 and meets the convergence standard. The final determination coefficient RSQ is 0.694, the stress value is 0.258, and the model fitting effect is good. Subsequently, output the results of Multidimensional scaling analysis. If the scattered points of two tows are close, there is a high degree of similarity in the homegarden plants between the two towns; otherwise, the degree of similarity is low.

### Redundancy analysis (RDA)

Redundancy analysis was conducted on the plant sources, plant functions, and characteristics of homegardens via Canono.5 [38]. The actual measurements of altitude, distance to the nearest market, distance to the county town, number of permanent residents in the household, average age of permanent residents, average years of education, and homegarden area were used (Additional file 1: Table S1). The number is used to indicate whether the family has knowledge of herbal medicine, with 0 indicating that households have almost no knowledge of herbal medicine and 1 indicating that households are herbalists or have knowledge of herbs. Detrended correspondence analysis (DCA) was performed before RDA was conducted. Determine the use of RDA based on the first axis size of the Length of Gradient in the DCA analysis results. When the result is less than 3, RDA based on linear models performs better than canonical correspondence analysis (CCA) based on unimodal models.

## Results

### Plant diversity in homegarden

**Fig. 3:**
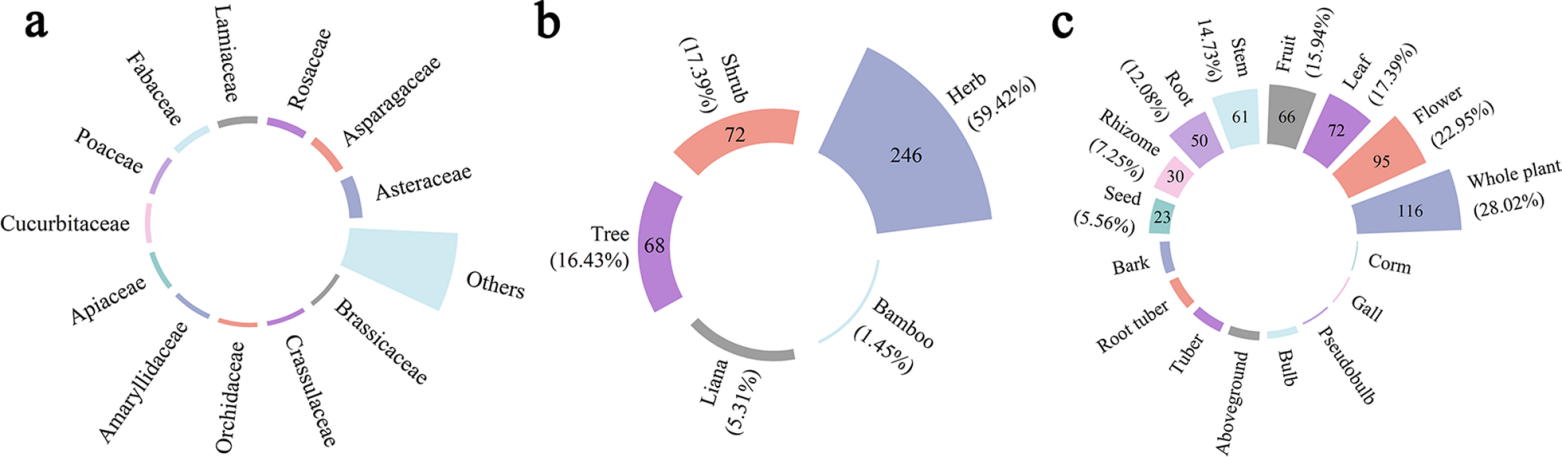
Plant distribution of the Laifeng Tujia ethnic communities. **a** Family distribution; **b** habit distribution; **c**use parts distribution

Many plant species are preserved in the homegarden of the Laifeng Tujia ethnic communities. Among the 243 homegardens surveyed, each encompassed between 17 and 121 species of plants, with an average of 40 species per household. A total of 414 homegarden plants from 114 families were recorded, and their scientific names, local names, family names, habits, sources, functions, utilization parts, utilization methods, and other information are provided in Table S4. These plants comprise three groups: ferns (4 species, 3 families, 0.97%), gymnosperms (11 species, 5 families, 2.66%), and angiosperms (399 species, 106 families, 96.38%). The 5 families with the greatest number of species were *Asteraceae* (29), *Asparagaceae* (19), *Rosaceae* (17), *Lamiaceae* (16), and *Fabaceae* (16) (Fig. 3a). Categorized by their habits, herbaceous plants (246, 59.42%) are the dominant component of homegardens, followed by shrubs (72, 17.39%), trees (68, 16.43%), and lianas (22, 5.31%) (Fig. 3b). Bamboo plants constitute the smallest number of species, with only 6. Multiple homegarden plants are fully utilized by the Laifeng Tujia ethnic community. Among them, the number of species that use whole plants (116, 28.02%) was the greatest, followed by flowers (95, 22.95%), leaves (72, 17.39%), fruits (66, 15.94%), and stems (61, 14.73%) (Fig. 3c).

### Sources and functions of homegarden plants

**Fig. 4:**
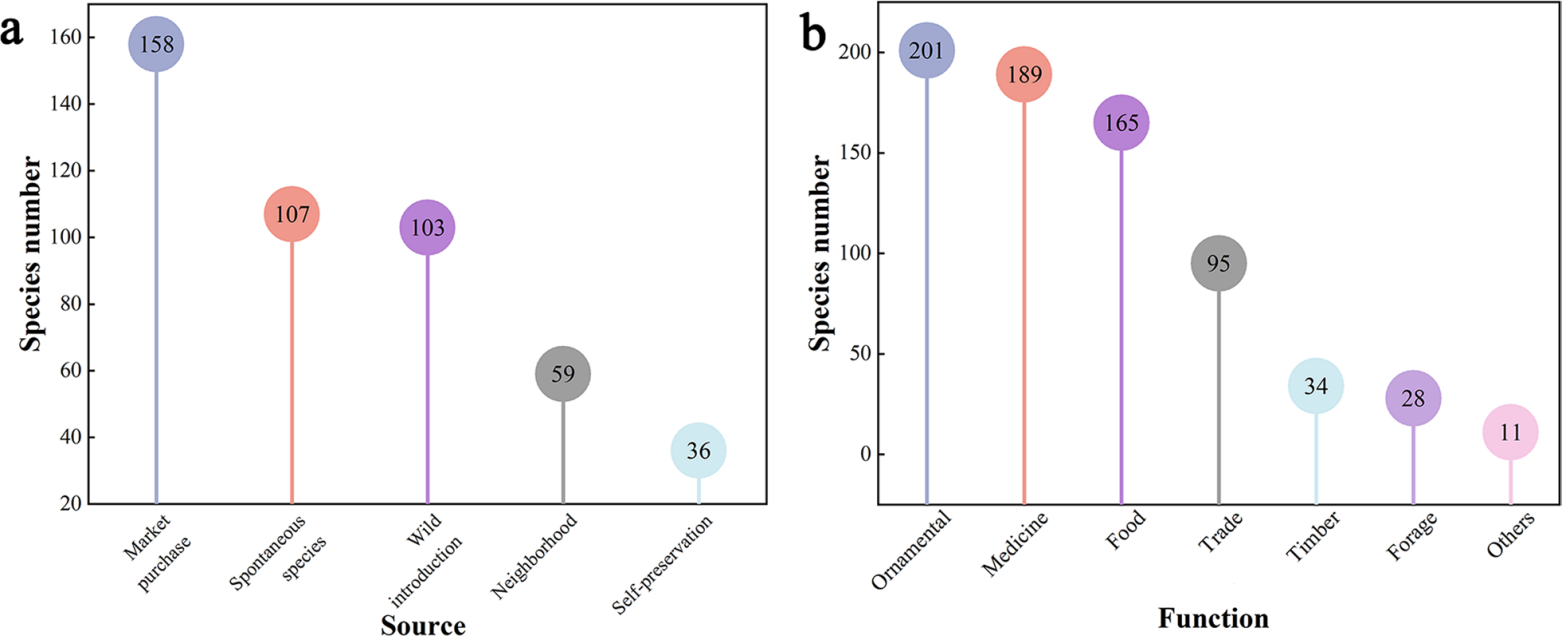
Plant sources and functions diversity in the homegarden of the Laifeng Tujia ethnic communities. **a** source; **b** function

The diverse sources of homegarden plants can be classified into five categories: market purchases (158), spontaneous species (107), wild introductions (103), neighborhood sharing (59), and self-preservation (36) (Fig. 4a). The market category includes three channels: purchasing at the market, online shopping, and local government unified procurement, with the highest number of species included. This is followed by spontaneous species, which refer to naturally growing species that are not artificially introduced and intentionally preserved in the homegarden.

The various functions and usage characteristics of homegarden plants can be divided into ornamentals (201), medicines (189), foods (165), trades (95), timber (34), forage (28), and others (11) (Fig. 4b). The high proportion of ornamental, medicinal, and food plants in the homegarden plants of the Laifeng Tujia ethnic community indicates that the homegarden provides an abundance of ornamental flowers, traditional medicinal plants, and edible fruits and vegetables for locals. The plants within these homegardens often possess multiple properties, indicating that a single plant usually performs multiple functions.

**Fig. 5:**
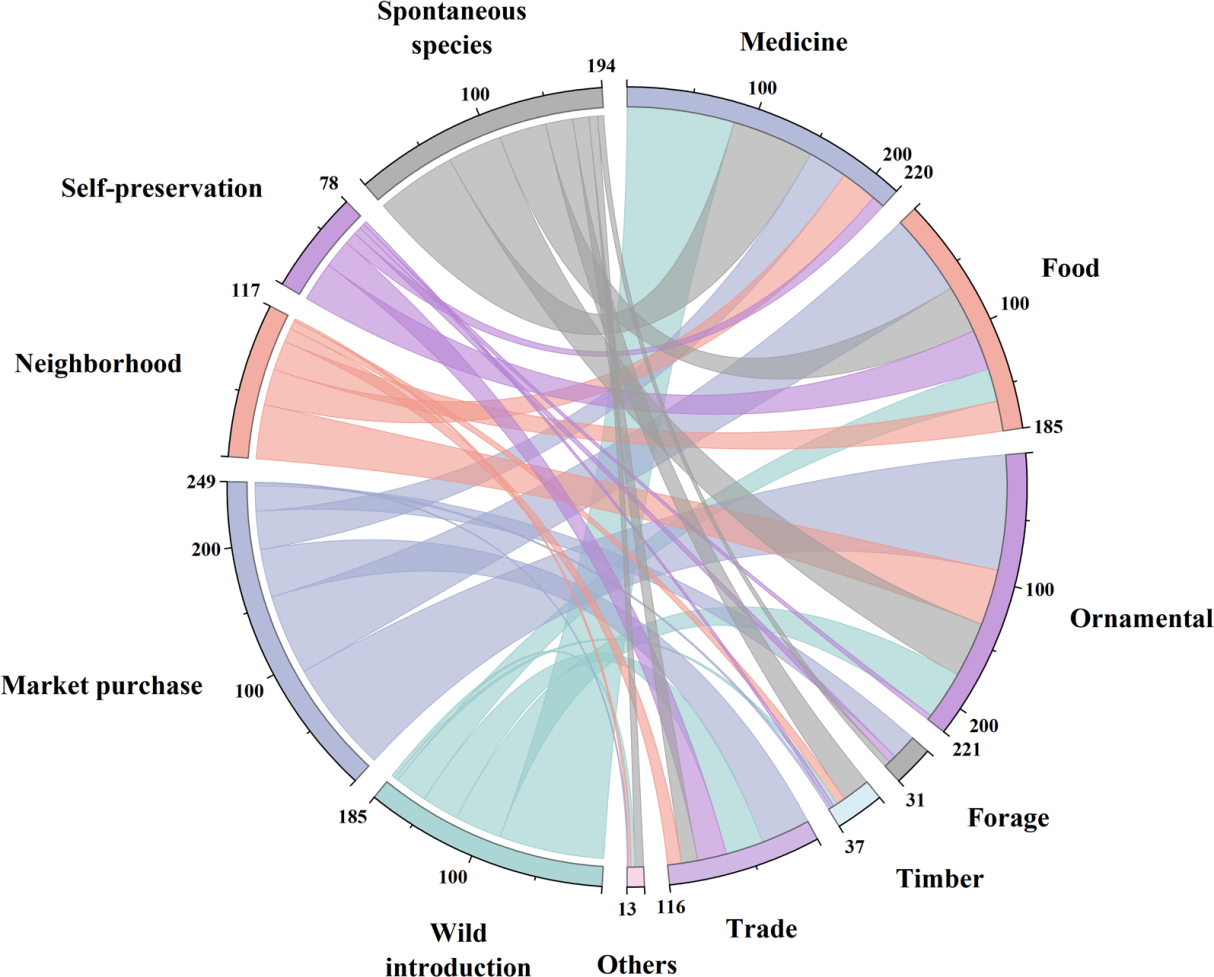
Chord diagram of the source and function association of plants in the homegarden of the Laifeng Tujia ethnic communities. Chord diagrams are used to demonstrate the correlation between the sources and functions of homegarden plants. The color of the chords is determined by the source of the homegarden plants. The thickness of the chords represents the strength of the connection between homegarden plants of different sources and functions

A correlation analysis of the sources and functions of homegarden plant species revealed that the main source of ornamentals is market purchases, including some grass flowers and potted plants (Fig. 5). Medicinal plants are sourced mainly from wild introductions and spontaneous species, consisting mainly of some folk herbs, some of which are also primary sources of trade plants. Foods are purchased predominantly from the market, primarily including seasonal vegetable seeds and seedlings.

#### Ornamentals

**Fig. 6:**
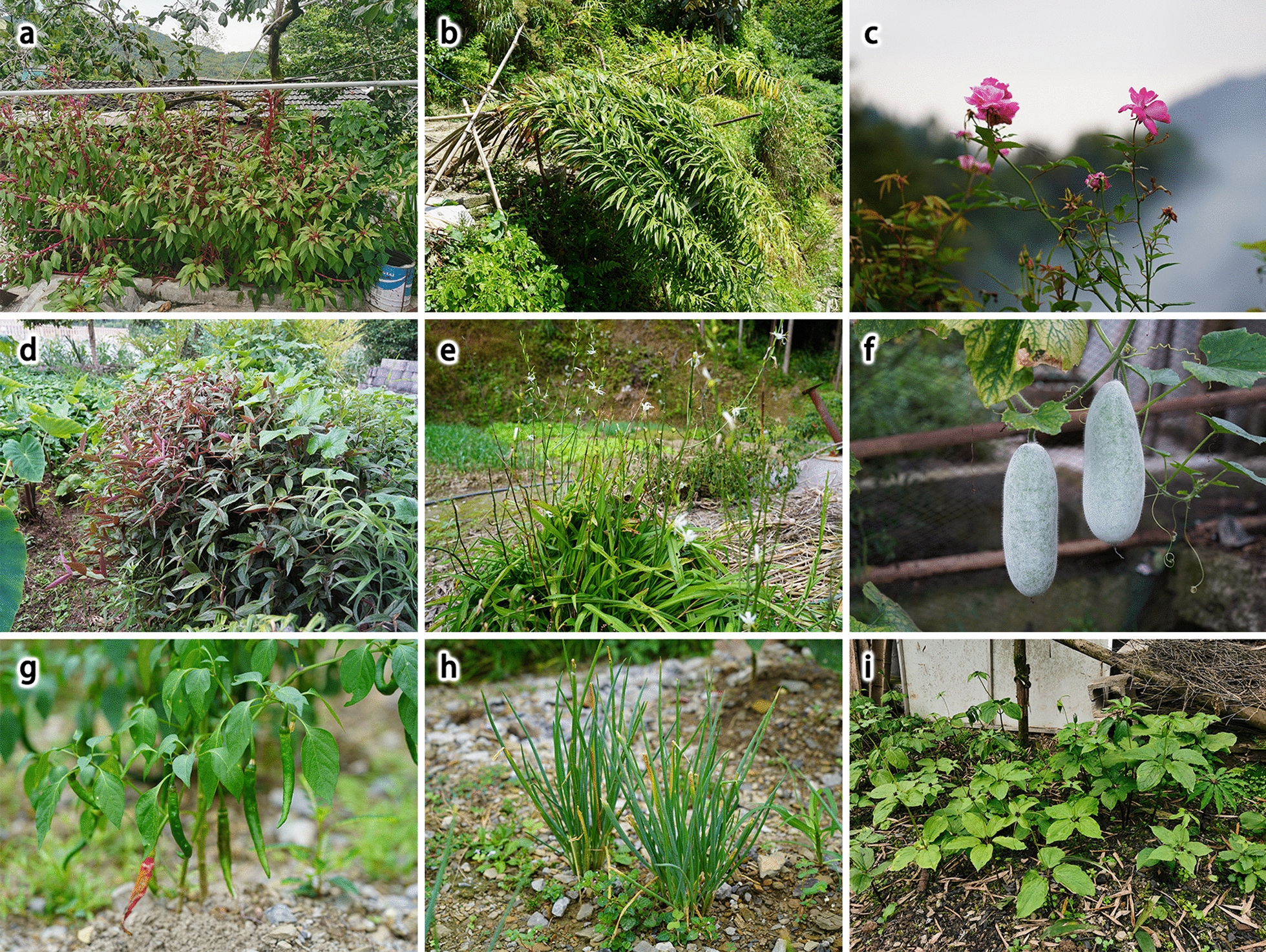
Some homegarden plants. **a** *Celosia cristata*; **b** *Polygonatum sibiricum*; **c** *Rosa chinensis*; **d** *Achyranthes longifolia*; **e** *Diuranthera major*; **f** *Benincasa hispida*; **g** *Capsicum annuum*; **h** *Allium fistulosum*; **i** *Paris fargesii*

The most prominent role of homegarden plants is providing ornamental beauty, a feature that contributes significantly to the aesthetic appeal of local homegardens. *Osmanthus fragrans*, *Lagerstroemia indica*, *Impatiens balsamina*, and *Celosia cristata* (Fig. 6a) represent the most prevalent ornamental species.

#### Medicines

Medicinal use constitutes another significant function of homegarden plants among the Laifeng Tujia ethnic communities. The locals maintain the belief that “all varieties of herbs can be medicinal, with the key lying in the knowledge of their proper use”. Our findings indicate that most households cultivate several to dozens of medicinal plants within homegardens.

*Polygonatum sibiricum*, a medicinal food plant, is cultivated in nearly every homegarden. Its rhizomes, when soaked in wine and consumed, can ameliorate lower back pain and exhibit a nourishing effect on muscle and bone fortification (Fig. 6b). Polysaccharides in *P. sibiricum* are essential components that have possessing biological activities, such as antioxidant, antiaging, antifatigue, and lipid-lowering activities [39]. Additionally, in periods of food scarcity, locals resort to roasting and consuming it. Presently, numerous medicinal herb merchants purchase it at a rate of approximately 16–20 RMB/kg, providing economic benefits to households.

*Rosa chinensis*, known for its highly ornamental flowers, is a beloved plant [40]. It serves both medicinal and ornamental purposes in the homegarden (Fig. 6c). According to local belief, its flowers and roots can be utilized to treat gynecological disorders, either through decoction or by soaking in wine for oral consumption. The medicinal effects of various *R. chinensis* varieties are also classified on the basis of their flower color. It is generally believed that *R. chinensis* with white flowers can address menstrual disorders, whereas varieties with red flowers are utilized to treat abnormal vaginal discharge.

Additionally, numerous medicinal plants, including *Achyranthes longifolia* (Fig. 6d), *Diuranthera major* (Fig. 6e), and *Eleutherococcus nodiflorus*, utilized for nourishment are present in the homegarden. These plants, which are typically employed in stewing meat, are widely accepted by the Laifeng Tujia people. Notably, local people also utilize certain vegetable species as traditional remedies. For instance, the roots of *Cucurbita moschata* can be used for the treatment of lymphatic inflammation, whereas the seeds of *Allium tuberosum* can be used to treat conditions such as hemorrhoids, toothache, and stomach ailments.

#### Foods

Among the homegardens of the Laifeng Tujia ethnic communities, vegetables, including *Raphanus sativus*, *Cucumis sativus*, and *Benincasa hispida* (Fig. 6f), constitute the primary edible plants. Plants in homegardens can provide an abundance of nutrients such as carbohydrates, vitamins, proteins, and dietary fiber, directly contributing to the sustenance of local inhabitants [41, 42]. Additionally, spice plants play a significant role in these homegardens. The Laifeng Tujia ethnic community states, “Using chili peppers as salt, with *Hezha* (a soy product stewed with vegetables), you can celebrate the Chinese New Year.” In the past, owing to limited transportation and resources, a variety of spice plants were cultivated in homegardens as substitutes for soy sauce, salt, and other seasonings. These include *Capsicum annuum* (Fig. 6g), *Zingiber officinale*, *Coriandrum sativum*, and *Allium chinense*, which were grown to enhance the flavor of the dishes. This practice persists today, forming an integral part of their dietary culture. This characteristic aligns with findings from research in the neighboring Larshan region in western Hunan [24].

#### Other categories

A significant portion of homegarden plants, encompassing edible and medicinal species, are used for trade. These plants serve as commodities for market trade or are procured by medicinal merchants to sustain or supplement livelihoods. Among the 34 species of homegarden plants used for timber, certain species, including *Cunninghamia lanceolata*, *Cupressus funebris*, *Toona sinensis*, and *Liquidambar formosana*, are utilized in the construction of characteristic Tujia stilt houses. Other varieties of homegarden plants are also employed in the fabrication of agricultural tools and as firewood. Local residents believe that plants edible to humans can also be consumed by livestock, resulting in various homegarden plants serving as forage for pigs, poultry, and livestock. Additionally, 11 species of homegarden plants are classified as other uses, predominantly encompassing cultural purposes, fence construction, and dyeing.

### RFC value analysis of homegarden plants

The *RFC* value range for homegarden plants of the Laifeng Tujia ethnic communities is between 0.004 and 0.951. 24 homegarden plants presented *RFC* values greater than 0.5, indicating their high significance. Among these, *Allium fistulosum* had the highest *RFC* value (0.951), followed by *Raphanus sativus* (0.914), *Brassica rapa* var. *chinensis* (0.897), *Brassica rapa* var. *glabra* (0.881), and *Cucurbita moschata* (0.881).

*A. fistulosum* is a common condiment among the Laifeng Tujia ethnic communities, and virtually every household cultivates it for its own use, which results in the highest *RFC* value (Fig. 6h). *R. sativus*, *B. rapa* var. *chinensis*, *B. rapa* var. *glabra*, *C. moschata*, *Vigna unguiculata* subsp. *Sesquipedalis*, *Lactuca sativa* var. *angustata*, and *Benincasa hispida* rank among the most commonly preserved homegarden plants within the Laifeng Tujia ethnic communities, all with high *RFC* values.

*Zea mays*, *Ipomoea batatas*, and *Solanum tuberosum* are prevalent food plants of the Laifeng Tujia ethnic communities in mountainous areas, and can also serve as forage for livestock and poultry. Certain households cultivate these plants in fields rather than within homegardens, leading to their *RFC* values not being as high as those of fruits and vegetables. Nonetheless, they still play an integral role in the locals’ livelihood.

Most herbal plants, such as *Eleutherococcus nodiflora*, *Mahonia belei*, and *Paris fargesii* (Fig. 6i), presented low *RFC* values. Homegardens that preserve these plant species often belong to households that practice herbalism or possess some knowledge of herbal medicine.

### Comparison of the homegarden plant species among the different towns

**Fig. 7:**
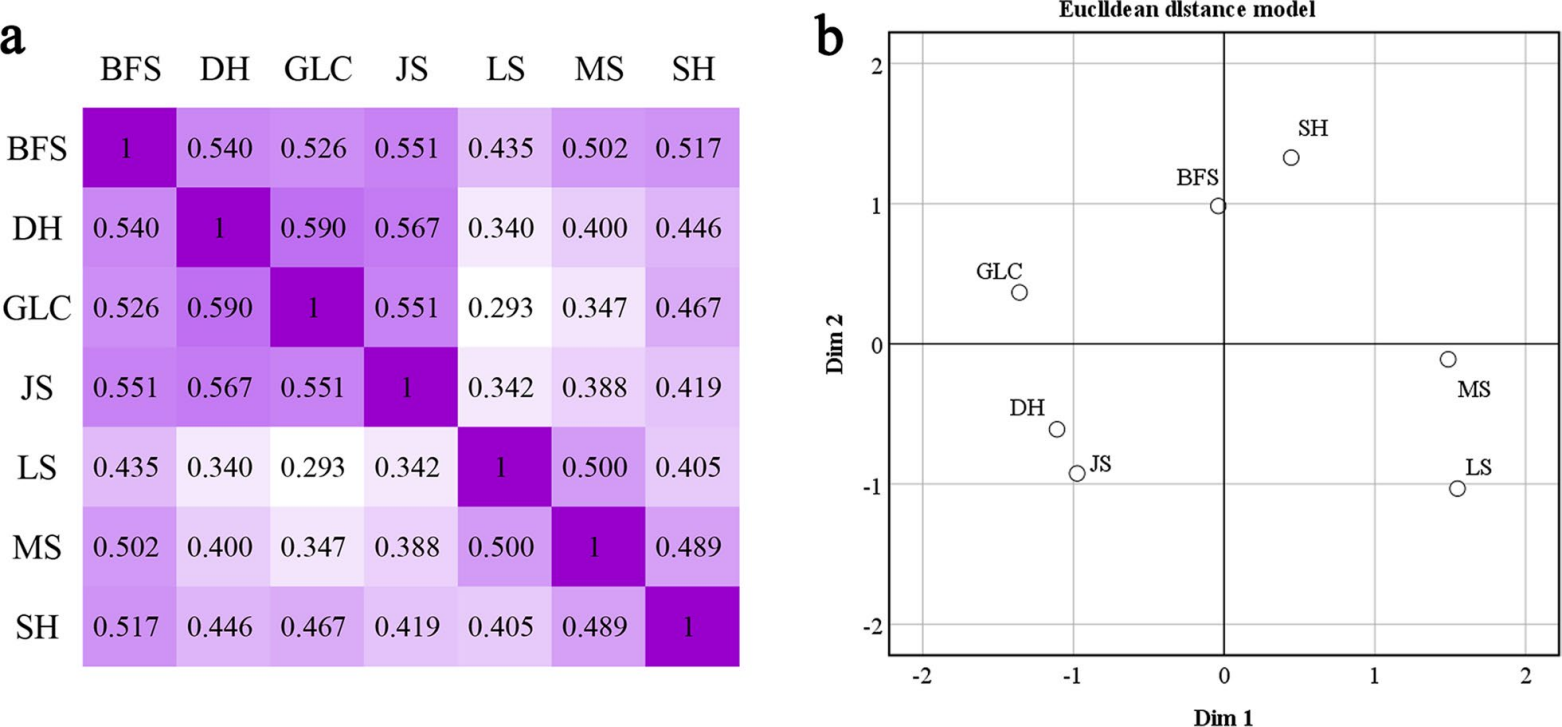
**a** Heatmap of Jaccard index for courtyard plants in different towns; **b** Multidimensional scaling based on Jaccard Distance (ALSCAL)

The Jaccard index results for homegarden plants across various towns are depicted in Fig. 7. According to the heatmap of the Jaccard index, the degree of similarity among homegarden plants in Geleche Town and Dahe Town was the most pronounced, at 0.590, whereas the similarity of homegarden plant species between Lvshui Town and Geleche Town was notably minimal, at 0.293 (Fig. 7a). In accordance with the multidimensional scaling outcomes of the Jaccard distance values, the seven towns are distributed across four quadrants (Fig. 7b). Dahe, Geleche, and Jiusi townships are situated in the elevated western region of Laifeng County, featuring proximate geographical positions. Manshui and Lvshui represent two neighboring townships situated in the eastern part of Laifeng County. Generally, the similarity among homegarden plants in towns with similar geographical locations is correspondingly elevated.

### Influences of homegarden characteristics on the sources and functions of homegarden plants

**Fig. 8:**
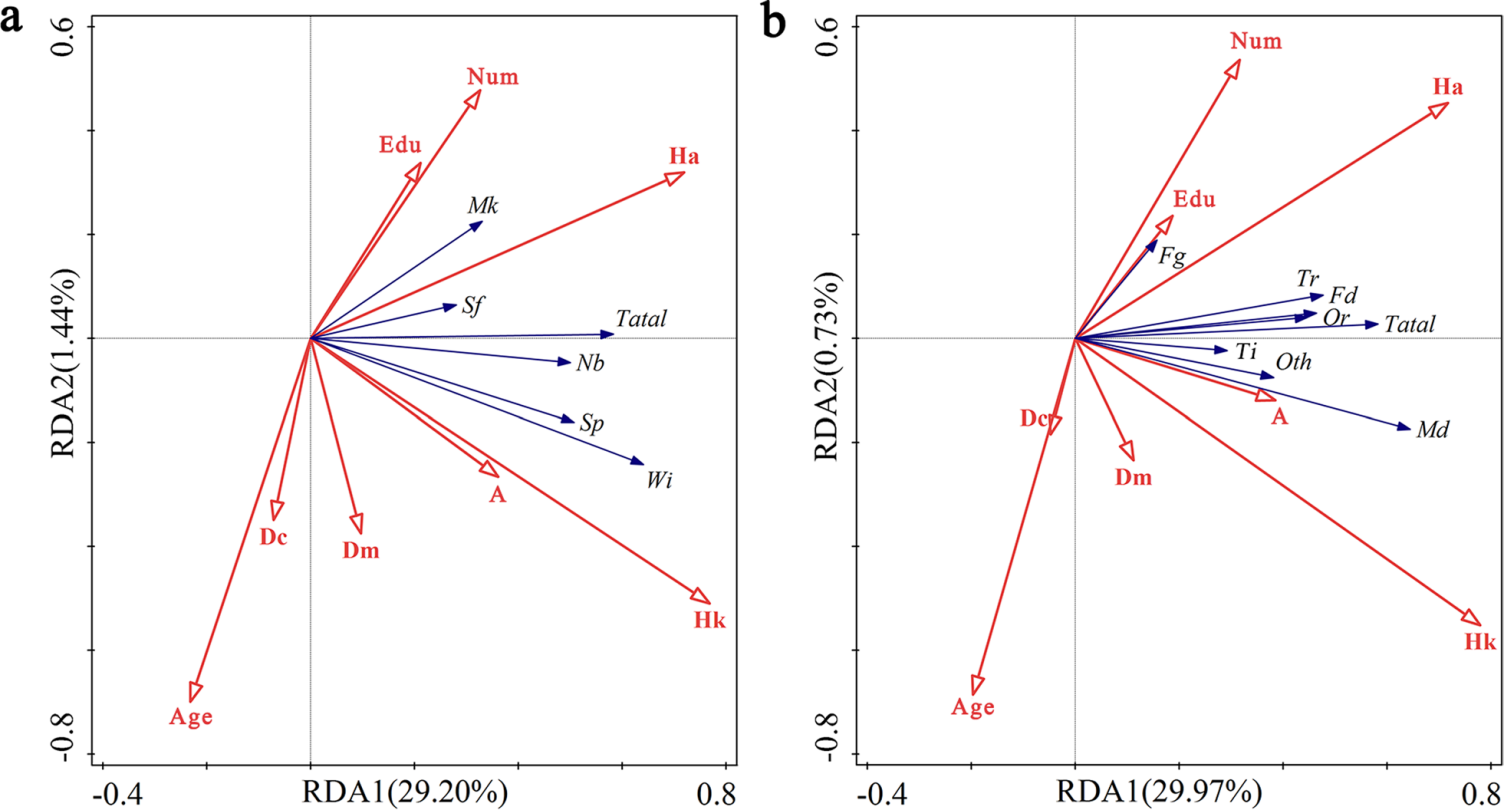
RDA ordination of plant sources and functions in the homegarden of the Laifeng Tujia ethnic communities. **a** source; **b** function *A* Altitude, *Dm* Distance to the nearest market, *Dc* Distance to the county town, *Num* Number of permanent residents in the household, *Age* Average age of permanent residents, *Edu* Average years of education, *Ha* Homegarden area, *Hk* Herbal medicine knowledge, *Wi* Wild introduction, *Mk* Market purchase, *Nb* Neighborhood sharing, *Sf* Self-preservation, *Sp* Spontaneous species, *Md* Medicine, *Fd* Food, *Or* Ornamental, *Fg* Forage, *Ti* Timber, *Tr* Trade, *Oth* Other

The DCA of the sources and functions of the homegarden plants revealed maximum gradient lengths of 0.6228 and 0.5264 along the four sorting axes, both of which were less than 3 (Table S2). Thus, RDA was selected to determine the sources and functions of homegarden plants. The RDA sorting results reveal that the eigenvalues of sorting axes 1 and sorting axes 2 for homegarden plant sources are 0.292 and 0.0144, respectively, accounting for 99.48% of the total eigenvalue (0.308) (Table S3). The eigenvalues of sorting axes 1 and sorting axes 2 of the homegarden plant functions are 0.2997 and 0.0073, respectively, reaching 98.90% of the total eigenvalue (0.3104) (Table S3). The first two sorting axes were selected as the RDA 2D sorting diagram (Fig. 8). The source and function of homegarden plants were significantly correlated with knowledge of herbal medicine and the homegarden area (*p* < 0.01) (Table 2). With respect to the source of homegarden plants, knowledge of herbal medicine and the homegarden area had the greatest impact on species from wild introductions (Fig. 8a). With respect to the function of homegarden plants, knowledge of herbal medicine and the homegarden area had the most significant impacts on the species used for medicine (Fig. 8b). Additionally, the average age of permanent residents has a notable negative correlation (*p* < 0.05) with the source and function of homegarden plants (Table 2).

**Table 2:** Explanation rate of influencing factors of RDA

	Characteristic	Explains %	Contribution %	pseudo-F	*P*
Source	Hk	17.6	57.1	51.5**	0.002
	Ha	10	32.4	33.1**	0.002
	Age	1.6	5.2	5.4*	0.014
	Dc	0.9	2.9	3.1	0.074
	A	0.5	1.5	1.5	0.204
	Num	0.2	0.5	0.5	0.534
	Dm	< 0.1	0.2	0.2	0.762
	Edu	< 0.1	0.2	0.2	0.786
Function	Hk	18.4	59.3	54.5**	0.002
	Ha	10	32.3	33.7**	0.002
	Age	1	3.3	3.4*	0.036
	A	0.7	2.3	2.4	0.08
	Dc	0.5	1.7	1.8	0.162
	Num	0.2	0.5	0.6	0.53
	Dm	0.1	0.3	0.4	0.662
	Edu	< 0.1	0.2	0.2	0.852

*Hk* Herbal medicine knowledge, *Ha* Homegarden area, *Age* Average age of permanent residents, *Dc* Distance to the county town, *A* Altitude, *Num* Number of permanent residents in the household, *Dm* Distance to the nearest market, *Edu* Average years of education

## Discussion

### Factors influencing of the plant diversity in homegardens

Previous studies have shown that plant diversity in homegardens is assumed to be determined by complex socio-economic and ecological factors as well as intrinsic characteristics of homegardens [18, 22, 43, 44]. We identified geographical features, household demographic characteristics, and homegarden area as the primary factors potentially affecting the composition of homegarden plants within the Laifeng Tujia ethnic community. Significant variations are observable in different homegardens. Overall, homegardens reflect the experiential and knowledge base of households. Herbal medicinal knowledge, in particular, is a crucial factor influencing the constitution of homegarden plants, especially support for medicinal plants (*p* < 0.01). Findings from southwestern Bangladesh demonstrate a positive correlation between the experience of the household head and the composition of homegarden plants, which aligns with our results [45]. Similar outcomes have been reported in studies conducted in Central Java, Indonesia [19]. Clearly, the comprehension of herbal knowledge echoes the overall wisdom and experience of households. In numerous households proficient in herbal medicine or with resident herbalists, we identified a variety of medicinal plants, which were obtained primarily from wild introductions, spontaneous species, and market purchases. In these homegardens, there is often a high degree of species diversity. In other studies, the owners of these home gardens are referred to as “masters” or “experts” [46–48]. These farmers are usually older and have rich traditional knowledge of utilizing homegarden plants.

The extent of land holdings can be viewed as a positive contributor to the richness of plant species in homegardens [45, 49]. For subsistence-oriented homegardens, the general hypothesis posits a positive correlation between the size of the homegarden and its species richness [50, 51]. In the present study, the homegarden area emerged as another significant factor influencing the composition of homegarden plants (*p* < 0.01), thereby substantiating this hypothesis. A larger homegarden area tends to harbor a greater diversity of plant species, particularly those introduced from the wild.

A positive correlation exists between the average age of the household resident population and the composition of homegarden plants (*p* < 0.05). Elderly family members, who possess greater life experience, may contribute significantly to the preservation of plants in the homegarden. This impact comes from multiple sources: previous studies on countries such as Nicaragua, Texas (USA), central Italia, and Benin have shown that adult and elderly people are more involved in gardening activities than young people [22, 52, 53]. Furthermore, the advancement in age results in the enrichment of knowledge through experience, which subsequently diversifies the assortment of plants in the homegardens of the elderly [48]. Secondly, with increasing age, the corresponding social networks become stronger, and the exchange of planting materials through these networks enhances the diversity of plants in homegardens, similar to the plant sources shared in neighborhood in our study [48]. Conversely, studies on homegardens in southern Ethiopia have demonstrated that the age of the household head did not significantly influence the diversity of homegarden plants [54]. There are also study findings supporting the notion that homegarden plant diversity is determined by the interaction between homegarden age and gardener age [22]. We infer that under a specific context, the diversity of species in homegardens increases with the age of the household residents, peaks, and then decreases. This inference warrants further in-depth study.

Educational level is also a widely discussed influencing factor. Research in Bangladesh shows that better education levels among households can increase the species richness of homegarden plants [18]. However, studies in Benin, Malaysia, and Uganda suggest that education level does not have a significant impact on plant diversity in homegardens [22, 23, 55]. Our current study revealed that, in addition to the average age of permanent residents, the number of household permanent residents and the average years of education did not significantly impact the composition of homegarden plants. On the one hand, in the survey area, the younger population has sought employment elsewhere, leaving behind primarily elderly individuals with lower levels of education. The average number of permanent residents and years of education are 2 and 4, respectively, which shows no significant variance. On the other hand, our study may not have comprehensively considered all pertinent factors, such as household income sources, per capita disposable income, and cultural background. These factors could have a nonlinear relationship with the composition and diversity of homegarden plants [48].

Concurrently, our study revealed that the altitude of homegardens did not significantly influence the composition of their plants. In the Yarlung Tsangpo Grand Canyon in southwestern China, considerable variation in the elevation of different homegardens of the Tsang-la (Motuo Menba) exists, and altitude is one of the most influential factors in the diversity and composition of homegarden plants [27]. Similar results have also been reported in studies in southern Ethiopia [56]. In Laifeng, the altitude variance among different homegardens is relatively minor, featuring a maximum difference of 596.3 m (Changtaiping Village in Geleche Town and Nanhe Village in Baifusi Town). The climatic conditions of homegardens at different altitudes are relatively similar. Furthermore, previous studies have suggested that proximity to markets may increase the richness of homegarden plants, particularly nonnative species [18, 54]. However, in our study, neither the distance to the nearest market town nor the county town significantly affected the composition of homegarden plants. In an era characterized by poor road conditions and resource scarcity, markets were the primary source for mountain residents to procure goods and trade commodities. With the implementation of China’s rural revitalization policy, improvements in infrastructure, and the widespread use of the internet, more remote mountainous households can now enjoy the convenience of online shopping. Numerous households in our survey reported acquiring horticultural plants through e-commerce, which to some extent positively impacts the diversity of garden flora. Additionally, some vendors operate small trucks laden with vegetables and fruits, essentially functioning as a “mobile market” between villages, which negatively impacts the diversity of homegarden plants. The impact of this liquidity on homegardens is also reflected in rural Myanmar [57]. Both methods facilitate closer proximity between families and markets, thereby diminishing the influence of geographical patterns on the composition and diversity of homegarden plants.

### Plant management and traditional culture inheritance

Homegardens serve not only as a vital part of agricultural ecosystems but also as significant social and cultural platforms for disseminating knowledge and experience [8]. The local management of homegarden plants not only fulfills their livelihood needs but also embodies a wealth of traditional culture and wisdom. Our survey revealed that the time spent on homegarden labor the locals varied considerably. Some locals stated that they would not spend excessive time in their homegardens, thus maintaining them primarily in a low-maintenance state. This management model reflects the deep understanding of the ecological environment by the Laifeng Tujia ethnic communities, emphasizing the principle of harmonious coexistence with nature.

Simultaneously, the Laifeng Tujia ethnic communities have incorporated a multitude of cultural symbols into the procedure of selecting and managing homegarden plants. For instance, they adhere to a creed known as “前不栽桑, 后不种桃” (*Qian bu zai sang, hou bu Zhong tao*), indicating that they avoid planting mulberry trees (*Morus alba*) at the front of the homegarden and peach trees (*Prunus persica*) at the rear. This avoidance stems from a linguistic association in Chinese, where “桑” sounds like “mourning” (*sang*), and “桃” resembles “escape” (*tao*), both of which are considered ill-omening. The aforementioned cultural symbols mirror the values and beliefs of locals, simultaneously fostering the sustained practice of homegarden plant management.

Furthermore, herbal plants are frequently found in closely situated homegardens or those belonging to families with herbal knowledge. The interchange of resources and cultural knowledge regarding these plants among locals serves as a beneficial tool for encouraging conservation [58, 59]. Such neighborhood sharing exemplifies their social network, and these homegarden plants embody their neighborhood relationships.

Notably, the primary focus of our survey is on villages that have managed to preserve their traditional culture and are surrounded by abundant forest resources. However, the rapid pace of economic development and land use has resulted in a reduction in homegarden planting areas in the surveyed region. Concurrently, mirroring trends in many Southeast Asian countries, homegardens are experiencing a substantial shift from subsistence-oriented to commercial-oriented [60]. The exodus of the younger population has resulted in labor shortages, further jeopardizing the management of homegarden plants and the preservation of related traditional cultural heritage. This phenomenon is global, as evidenced in research in countries such as Colombia and Brazil [61, 62]. The younger generation shows little interest in it, and the traditional knowledge about homegarden plants left by the older generation has not been inherited. The serious threat to traditional knowledge has affected the future sustainability of traditional knowledge related to homegarden plant management. In light of these circumstances, the negative implications of these changes warrant serious consideration.

## Conclusion

The present study targeted 25 villages within the Laifeng Tujia ethnic communities, which are located in the hinterland of the Wuling mountain area, to carry out an ethnobotanical survey of their homegarden plants. A total of 114 families and 414 species of plants were recorded in 243 homegardens, which play crucial roles in maintaining the balance of the homegarden ecosystem and supporting the daily livelihoods of the Laifeng Tujia ethnic communities. The homegarden plants originate from a variety of sources, primarily market purchases, spontaneous species, wild introductions, neighborhood sharing, and self-preservation. They are used predominantly for various functions, including ornamental, medicinal, edible, trade, timber, forage, and others. We postulate that having knowledge of herbal medicine and the extent of the homegarden area might be the most crucial factors influencing the composition and diversity of homegarden plants, with the average age of permanent residents also exerting an impact. The homegarden practices of the Laifeng Tujia ethnic communities and their management of homegarden plants carry their traditional agricultural knowledge and wisdom. In view of future developments, efforts should be made to understand, safeguard, and disseminate the traditional knowledge associated with homegardens, as they are paramount for the preservation of local agricultural biodiversity and the promotion of sustainable development.

Based on the findings of our research, we propose the following recommendations:

1. Establishment of a homegarden plant database: Digitally store information on homegarden plants and develop a database system that is easy to query and update.
2. Promotion of traditional knowledge education and training: The organization of lectures and training sessions on homegarden plants and traditional knowledge is crucial in enhancing residents’ conservation awareness. Further, it is essential to encourage the younger generation to learn and inherit traditional knowledge to prevent its loss.
3. Implementation of the homegarden plant protection plan: There should be an establishment of a special fund to support the planting, protection, and restoration of homegarden plants.
4. Strengthening of community participation and cooperation: There should be organization of community activities to motivate the residents’ participation in the protection and planting of homegarden plants. A community protection group should be established to jointly supervise and maintain the plant resources in the homegardens.

## Supplementary Information

Additional file 1.

Additional file 2.

Additional file 3.

## Data Availability

All data generated or analyzed during this study were included in this published article (along with the supplementary files).

## Abbreviations

FC: Frequency of citation
RFC: Relative frequency of citation
J: Jaccard index
JD: Jaccard distance
DCA: Detrended correspondence analysis
RDA: Redundancy analysis
