# Supplementary material for: Ethnobotanical study on factors influencing plant composition and traditional knowledge in homegardens of Laifeng Tujia ethnic communities, the hinterland of the Wuling mountain area, central China
Source: J Ethnobiol Ethnomed. 2024 Dec 2;20:103. doi: 10.1186/s13002-024-00742-4 (PMC11610127; doi:10.1186/s13002-024-00742-4)
Supplement: Supplementary file 2 — Additional file 2. [file 13002_2024_742_MOESM2_ESM.docx]

Table S2 Eigenvalues of the ordination axis of DCA

|  | Source | | | | Function | | | |
| --- | --- | --- | --- | --- | --- | --- | --- | --- |
| Statistic | Axis 1 | Axis 2 | Axis 3 | Axis 4 | Axis 1 | Axis 2 | Axis 3 | Axis 4 |
| Eigenvalues | 0.0292 | 0.0112 | 0.0032 | 0.0017 | 0.0324 | 0.0111 | 0.0044 | 0.0019 |
| Explained variation (cumulative) | 64.51 | 89.22 | 96.39 | 100.21 | 53.27 | 71.54 | 78.76 | 81.83 |
| Gradient length | 0.76 | 0.69 | 0.42 | 0.47 | 0.58 | 0.64 | 0.46 | 0.39 |
| Pseudo-canonical correlation (suppl.) | 0.6228 | 0.3273 | 0.3825 | 0.2743 | 0.5264 | 0.2438 | 0.2145 | 0.227 |

Table S3 Eigenvalues of the ordination axis of RDA

|  | Source | | | | Function | | | |
| --- | --- | --- | --- | --- | --- | --- | --- | --- |
| Statistic | Axis 1 | Axis 2 | Axis 3 | Axis 4 | Axis 1 | Axis 2 | Axis 3 | Axis 4 |
| Eigenvalues | 0.292 | 0.0144 | 0.001 | 0.0006 | 0.2997 | 0.0073 | 0.0025 | 0.0009 |
| Explained variation (cumulative) | 29.2 | 30.64 | 30.74 | 30.8 | 29.97 | 30.7 | 30.95 | 31.05 |
| Pseudo-canonical correlation | 0.5879 | 0.3765 | 0.2635 | 0.1817 | 0.6014 | 0.3114 | 0.2027 | 0.2599 |
| Explained fitted variation (cumulative) | 94.74 | 99.41 | 99.73 | 99.93 | 96.46 | 98.81 | 99.61 | 99.91 |
