# Supplementary material for: Ethnobotanical study on factors influencing plant composition and traditional knowledge in homegardens of Laifeng Tujia ethnic communities, the hinterland of the Wuling mountain area, central China
Source: J Ethnobiol Ethnomed. 2024 Dec 2;20:103. doi: 10.1186/s13002-024-00742-4 (PMC11610127; doi:10.1186/s13002-024-00742-4)
Supplement: Supplementary file 3 — Additional file 3. [file 13002_2024_742_MOESM3_ESM.docx]

Table 4 Inventory of homegarden plants in the Laifeng Tujia ethnic communities

| **Scientific name** | **Local name** | **Family name** | **Habit** | **Source** | **Function** | **Use part** | **Use method** | ***F*** | ***RFC*** | **Voucher number** |
| --- | --- | --- | --- | --- | --- | --- | --- | --- | --- | --- |
| *Asplenium prolongatum* Hook. |  | Aspleniaceae | Herb | Wild | Medicine | Whole plant | Soaked in wine or mashed and external applied to promote blood circulation and dispel stasis, to treat traumatic injury | 1 | 0.004 | PLF311005 |
| *Ophioglossum reticulatum* L. | Yi zi jian | Ophioglossaceae | Herb | Wild | Medicine | Whole plant | Soaked in wine or mashed and external applied to treat snakebite, blister, stomachache | 3 | 0.012 | LFE40414 |
| *Drynaria roosii* Nakaike | Gu sui bu | Polypodiaceae | Herb | Spontaneous species | Medicine | Rhizome | Decoction and orally taken to treat traumatic injury and cataclasis, to promote blood circulation and dispel stasis | 1 | 0.004 | PLF408022 |
| *Lepisorus fortunei* (T. Moore) C. M. Kuo | Qi xing jian | Polypodiaceae | Herb | Spontaneous species | Medicine | Whole plant | Decoction and orally taken to treat cough and cold, gynecopathy, lithiasis | 3 | 0.012 | LFE40149 |
| *Cryptomeria japonica* (Thunb. ex L. f.) D. Don | Sa su | Cupressaceae | Tree | Spontaneous species | Ornamental, timber | Stem | Ornamental; firewood | 23 | 0.095 | PLF408121 |
| *Cunninghamia lanceolata* (Lamb.) Hook. | Sa su | Cupressaceae | Tree | Spontaneous species | Medicine, ornamental, timber, trade | Leaf, stem, bark | Leaf: decoction and orally taken to treat cough and cold, as antidiarrheal; tender stem and leaf: mashed and external applied to treat snakebite and centipede bite, medicinal bath to ward off evil spirits on the Dragon Boat Festival; bark: medicinal bath to treat blister; stem: materials for construction, furniture, tools, and bee buckets, firewood; ornamental; trade | 43 | 0.177 | PLF406008 |
| *Cupressus funebris* Endl. | Bai zi su | Cupressaceae | Tree | Spontaneous species | Medicine, ornamental, timber, others | Stem, leaf, seed | Stem: firewood, construction material; leaf: burned for smoking bacon, repel mosquito; seed: soaked in wine and orally taken or external applied to dispel wind and eliminate dampness; ornamental | 2 | 0.008 | PLF408110 |
| *Juniperus chinensis* Roxb. | Bai zi su | Cupressaceae | Tree | Spontaneous species | Medicine, ornamental, timber, others | Stem, leaf, seed | Stem: firewood, construction material; leaf: burned for smoking bacon, repel mosquito; seed: soaked in wine and orally taken or external applied to dispel wind and eliminate dampness; ornamental | 1 | 0.004 | PLF408113 |
| *Juniperus squamata* Buch.-Ham. ex D. Don |  | Cupressaceae | Shrub | Market | Ornamental | Whole plant | Ornamental | 9 | 0.037 | PLF408055 |
| *Metasequoia glyptostroboides* Hu & W. C. Cheng | Sui sa | Cupressaceae | Tree | Market | Ornamental | Whole plant | Ornamental | 2 | 0.008 | PLF408082 |
| *Platycladus orientalis* (L.) Franco | Bai zi su | Cupressaceae | Tree | Spontaneous species | Medicine, ornamental, timber, others | Stem, leaf, seed | Stem: firewood, construction material; leaf: burned for smoking bacon, repel mosquito; seed: soaked in wine and orally taken or external applied to dispel wind and eliminate dampness; ornamental | 7 | 0.029 | PLF408059 |
| *Cycas revoluta* Thunb. | Tie su | Cycadaceae | Tree | Market | Ornamental | Whole plant | Ornamental | 15 | 0.062 | PLF406005 |
| *Ginkgo biloba* L. | Bai guo su | Ginkgoaceae | Tree | Market | Medicine, food, ornamental | Leaf, seed | Leaf: decoction and orally taken to treat heart disease and enteritis stomachache; seed: stir-fried; ornamental | 26 | 0.107 | LFE40471 |
| *Podocarpus macrophyllus* (Thunb.) Sweet | Luo han song | Podocarpaceae | Tree | Market | Ornamental | Whole plant | Ornamental | 15 | 0.062 | PLF408017 |
| *Taxus wallichiana* var. *mairei* (Lemée & H. Lév.) L. K. Fu & Nan Li | Hong fei | Taxaceae | Tree | Wild | Medicine, food, ornamental, timber | Fruit, stem | Fruit: eaten directly or soaked in wine and eaten, soaked in wine and orally taken to treat cancer, lithiasis; stem: furniture material; ornamental | 61 | 0.251 | LFE30067 |
| *Peristrophe japonica* (Thunb.) Bremek. | Qing ce lan | Acanthaceae | Herb | Neighborhood | Medicine | Leaf | Boiled or stir-fried with eggs and eaten to treat headache | 5 | 0.021 | PLF408054 |
| *Strobilanthes dimorphotricha* Hance | Hong ce lan | Acanthaceae | Herb | Wild | Medicine | Leaf | Boiled or stir-fried with eggs and eaten to treat headache | 3 | 0.012 | LFE40440 |
| *Acorus calamus* L. | Chang pu | Acoraceae | Herb | Wild | Medicine, others | Rhizome, leaf | Rhizome: decoction and orally taken to treat dysentery; leaf: as a door hanging to ward off evil spirits on the Dragon Boat Festival | 1 | 0.004 | LFE40370 |
| *Acorus gramineus* Soland. | Sui cang pu, san nai | Acoraceae | Herb | Wild, neighborhood | Medicine, food, trade, others | Leaf, flower, rhizome, whole plant | Leaf: as spice, stewed with meat and eaten; flower: external applied to treat burn and scald; rhizome: decoction and orally taken to treat cough and cold and stomachache, to dispel wind and eliminate dampness, to relieve pain, purchased by medicinal herb merchants; whole plant: as a door hanging to ward off evil spirits on the Dragon Boat Festival | 16 | 0.066 | LFE40167 |
| *Actinidia chinensis* Planch. | Yang tao | Actinidiaceae | Liana | Market | Food | Fruit | Eaten directly or soaked in wine and eaten | 18 | 0.074 | LFE40089 |
| *Liquidambar formosana* Hance | Feng su | Altingiaceae | Tree | Spontaneous species | Medicine, ornamental, timber | Leaf, bark, fruit | Soaked in wine and orally taken or external applied, to dispel wind and eliminate dampness, to treat traumatic injury; medicinal bath to ward off evil spirits on the Dragon Boat Festival; construction material, firewood; ornamental | 4 | 0.016 | PLF311008 |
| *Achyranthes longifolia* (Makino) Makino | Hong liu ke xi | Amaranthaceae | Herb | Wild | Medicine, food, ornamental | Root | Mashed and external applied, decoction and orally taken or external applied, to dispel wind and eliminate dampness, to treat traumatic injury, hypertension, gynecopathy, to relieve pain; stewed with meat and eaten to enrich blood reinforce Qi and to treat strengthen musle and bone; ornamental | 41 | 0.169 | LFE40359 |
| *Amaranthus caudatus* L. | Han cai | Amaranthaceae | Herb | Neighborhood | Ornamental | Whole plant | Ornamental | 2 | 0.008 | PLF408073 |
| *Amaranthus tricolor* L. | Han cai | Amaranthaceae | Herb | Self-preservation | Food, trade | Tender stem and leaf | Stir-fried and eaten; trade it at the market | 72 | 0.296 | PLF408085 |
| *Amaranthus hypochondriacus* L. | Ji guan hua | Amaranthaceae | Herb | Neighborhood | Ornamental | Whole plant | Ornamental | 2 | 0.008 | PLF408101 |
| *Beta vulgaris* var. *cicla* L. | Liu pi cai | Amaranthaceae | Herb | Market | Food | Leaf | Stir-fried and eaten | 57 | 0.235 | PLF408094 |
| *Celosia cristata* L. | Ji guan hua | Amaranthaceae | Herb | Neighborhood | Medicine, ornamental | Flower, seed | Decoction or soaked in wine and orally taken to treat irregular menses and abnormal leucorrhea; ornamental | 61 | 0.251 | LFE40044 |
| *Spinacia oleracea* L. | Ce gen cai | Amaranthaceae | Herb | Market | Food, trade | Whole plant | Stir-fried and eaten; trade it at the market | 84 | 0.346 | PLF311019 |
| *Allium cepa* L. | Yang cong | Amaryllidaceae | Herb | Market | Food | Bulb | Stir-fried and eaten or as spice | 2 | 0.008 | PLF404015 |
| *Allium chinense* G. Don | Jiao tou | Amaryllidaceae | Herb | Self-preservation | Food | Bulb | Made cold dishes or as pickle and eaten | 193 | 0.794 | PLF408092 |
| *Allium fistulosum* L. | Huo cong | Amaryllidaceae | Herb | Self-preservation | Food | Whole plant | As spice | 231 | 0.951 | PLF408098 |
| *Allium hookeri* Thwaites | Da jiu cai | Amaryllidaceae | Herb | Neighborhood | Food | Aboveground | Stir-fried and eaten | 7 | 0.029 | PLF408047 |
| *Allium sativum* L. | Da suan | Amaryllidaceae | Herb | Self-preservation | Food | Bulb, leaf | Bulb: as spice or as pickle and eaten; leaf: as spice or stir-fried and eaten | 207 | 0.852 | PLF310009 |
| *Allium tuberosum* Rottler ex Spreng. | Jiu cai | Amaryllidaceae | Herb | Neighborhood | Medicine, food, trade | Aboveground, seed | Aboveground: stir-fried, trade it at the market; seed: decoction or external applied to treat hemorrhoid, grounded into powder and orally taken with water to treat toothache and stomachache | 178 | 0.733 | PLF408035 |
| *Hippeastrum striatum* Herb. |  | Amaryllidaceae | Herb | Market | Ornamental | Flower | Ornamental | 31 | 0.128 | PLF404010 |
| *Lycoris aurea* (L'Hér.) Herb. | Si suan | Amaryllidaceae | Herb | Wild | Medicine, ornamental | Bulb, flower | Bulb: grounded into powder and external applied as narcotic; flower: ornamental | 2 | 0.008 | PLF408097 |
| *Lycoris radiata* (L'Hér.) Herb. | Si suan | Amaryllidaceae | Herb | Wild | Medicine, ornamental | Bulb, flower | Bulb: grounded into powder and external applied as narcotic; flower: ornamental | 4 | 0.016 | PLF311007 |
| *Zephyranthes candida* (Lindl.) Herb. | Cong hua | Amaryllidaceae | Herb | Neighborhood | Ornamental | Flower | Ornamental | 6 | 0.025 | PLF408053 |
| *Zephyranthes carinata* Herb. | Cong hua | Amaryllidaceae | Herb | Neighborhood | Ornamental | Flower | Ornamental | 10 | 0.041 | PLF408124 |
| *Rhus chinensis* Mill. | Bei zi su | Anacardiaceae | Tree | Spontaneous species | Trade, others | Gall | Purchased by medicinal herb merchants; soaked in water to dye white silk for the light red color | 2 | 0.008 | PLF408095 |
| *Angelica decursiva* (Miq.) Franch. & Sav. | Ya jio qi, tu dang gui | Apiaceae | Herb | Wild | Medicine | Root | Decoction and orally taken to dispel wind and eliminate dampness, to treat traumatic injury; mashed and external applied to treat blister | 7 | 0.029 | LFE40301 |
| *Anthriscus sylvestris* (L.) Hoffm. | Tu tian qi | Apiaceae | Herb | Wild | Medicine | Root | Decoction and orally taken to treat traumatic injury, rectify Qi and relieve pain | 2 | 0.008 | PLF406029 |
| *Apium graveolens* L. | Qin cai | Apiaceae | Herb | Market | Food | Petiole | Stir-fried and eaten | 34 | 0.14 | PLF406020 |
| *Bupleurum marginatum* Wall. ex DC. | Cai fu | Apiaceae | Herb | Wild | Medicine | Whole plant | Decoction and orally taken to treat cough and cold, hepatitis, lithiasis | 5 | 0.021 | LFE40367 |
| *Conioselinum anthriscoides* (H. Boissieu) Pimenov & Kljuykov | Gao ben | Apiaceae | Herb | Wild | Medicine | Whole plant | Decoction and orally taken to rectify Qi and relieve pain | 1 | 0.004 | PLF404016 |
| *Coriandrum sativum* L. | Yan sui | Apiaceae | Herb | Market, self-preservation | Medicine, food, trade | Stem, leaf | Decoction and orally taken to treat eczema; as spice; trade it at the market | 175 | 0.72 | PLF310010 |
| *Cryptotaenia japonica* Hassk. | Ya jio ban | Apiaceae | Herb | Spontaneous species | Medicine, food, forage | Tender stem and leaf, whole plant | Tender stem and leaf: stir-fried or boiled and eaten; whole plant: forage for pigs, decoction and orally taken to treat detumescence | 17 | 0.07 | LFE30088 |
| *Daucus carota* var. *sativus* Hoffm. | Fu luo bo | Apiaceae | Herb | Market | Food, trade | Root | Stir-fried or boiled and eaten; trade it at the market | 71 | 0.292 | PLF310011 |
| *Foeniculum vulgare* Mill. | Xiao hui xiang | Apiaceae | Herb | Market | Food | Tender stem and leaf | Stir-fried and eaten or as spice | 20 | 0.082 | PLF408075 |
| *Heracleum moellendorffii* Hance | Be zi | Apiaceae | Herb | Wild | Medicine, trade | Root | Decoction and orally taken to treat heart disease, colpomycosis, cyst of uterus, to relieve pain; purchased by medicinal herb merchants | 1 | 0.004 | PLF403005 |
| *Ligusticum sinense* *'Chuanxiong'* | Cuan xiong | Apiaceae | Herb | Market, neighborhood | Medicine, trade | Rhizome | Decoction and orally taken to treat heart disease, stomachache, hiccups, to rectify Qi and relieve pain; soaked in wine and orally taken or external applied to relieve arthralgia, to dispel wind and eliminate dampness, to treat traumatic injury and hypertension; purchased by medicinal herb merchants | 11 | 0.045 | LFE40213 |
| *Oenanthe javanica* (Blume) DC. | Ye qin cai | Apiaceae | Herb | Spontaneous species | Food, forage | Tender stem and leaf | Stir-fried and eaten; forage for pigs | 4 | 0.016 | LFE40032 |
| *Catharanthus roseus* (L.) G. Don |  | Apocynaceae | Shrub | Market | Ornamental | Flower | Ornamental | 1 | 0.004 | PLF408056 |
| *Ilex pernyi* Franch. | Liu guo qi | Aquifoliaceae | Shrub | Wild | Medicine | Root, stem | Soaked in wine and orally taken to treat traumatic injury | 2 | 0.008 | LFE40228 |
| *Amorphophallus konjac* K. Koch | Mo ru | Araceae | Herb | Self-preservation, spontaneous species | Food, trade | Tuber | Starch extraction; trade it at the market | 115 | 0.473 | LFE30002 |
| *Arisaema erubescens* (Wall.) Schott | Se mo ru | Araceae | Herb | Spontaneous species | Medicine | Tuber | Mashed and external applied to treat blister; stir fried paste and orally taken to moisten lung and relieve cough | 2 | 0.008 | LFE40221 |
| *Colocasia antiquorum* Schott | Guang huo | Araceae | Herb | Neighborhood | Food, ornamental | Petiole, whole plant | Petiole: stir-fried and eaten; whole plant: ornamental | 10 | 0.041 | PLF408001 |
| *Colocasia esculenta* (L.) Schott | Yu tou | Araceae | Herb | Self-preservation, spontaneous species | Food, trade | Tuber | Boiled and eaten; trade it at the market | 119 | 0.49 | PLF404003 |
| *Leucocasia gigantea* (Blume) Schott | Lang dou, wu dou | Araceae | Herb | Spontaneous species | Medicine, ornamental | Flower, Rhizome | Ground into powder external applied to relieve pain, as narcotic; ornamental | 1 | 0.004 | PLF311003 |
| *Pinellia pedatisecta* Schott | Da san bu tiao | Araceae | Herb | Neighborhood | Medicine, trade | Tuber | Mashed and external applied to treat traumatic injury, to disperse swelling and relieve pain; purchased by medicinal herb merchants | 1 | 0.004 | PLF406003 |
| *Pinellia ternata* (Thunb.) Ten. ex Breitenb. | San bu tiao | Araceae | Herb | Self-preservation | Medicine, trade | Tuber | Mashed and external applied to treat snakebite, centipede bite, traumatic injury, to disperse swelling and relieve pain, to dispel wind and eliminate dampness; wrap and orally taken to eliminate phlegm and relieve cough, to treat hiccups; purchased by medicinal herb merchants | 8 | 0.033 | LFE40182 |
| *Aralia elata* (Miq.) Seem. | Ci luo bu | Araliaceae | Tree | Spontaneous species | Food | Tender stem and leaf | Stir-fried or made cold dishes and eaten | 1 | 0.004 | LFE40212 |
| *Eleutherococcus nodiflorus* (Dunn) S. Y. Hu | Wu jia pi | Araliaceae | Shrub | Wild | Medicine, food | Tender stem and leaf, root bark | Tender stem and leaf: stir-fried or make soup and eaten; root bark: stewed with meat and eaten to enrich blood reinforce Qi, decoction and orally taken, or soaked in wine and orally taken or external applied, to dispel wind and eliminate dampness, to treat traumatic injury, pulmonary tuberculosis, gynecopathy | 17 | 0.07 | LFE40091 |
| *Eleutherococcus trifoliatus* (L.) S. Y. Hu | San jia pi | Araliaceae | Shrub | Wild, spontaneous species | Medicine | Root bark | Soaked in wine and orally taken to treat traumatic injury and to dispel wind and eliminate dampness | 3 | 0.012 | LFE40449 |
| *Hedera nepalensis* var. *sinensis* (Tobler) Rehder | San guo hong | Araliaceae | Shrub | Spontaneous species | Medicine | Whole plant | Medicinal bath to dispel wind and eliminate dampness and to treat blister | 4 | 0.016 | LFE40128 |
| *Heptapleurum arboricola* Hayata |  | Araliaceae | Shrub | Market | Ornamental | Whole plant | Ornamental | 1 | 0.004 | PLF408030 |
| *Heptapleurum bodinieri* H. Lév. | Qi guo hong | Araliaceae | Tree | Spontaneous species | Medicine | Whole plant | Decoction and orally taken or medicinal bath to treat postpartum body pain, blister, traumatic injury, to dispel wind and eliminate dampness | 2 | 0.008 | LFE40339 |
| *Hydrocotyle sibthorpioides* Lam. | Yu dian cao | Araliaceae | Herb | Spontaneous species | Medicine, food | Aboveground | Stir-fried with eggs and eaten to improve eyesight | 5 | 0.021 | LFE40084 |
| *Tetrapanax papyrifer* (Hook.) K. Koch | Da tong | Araliaceae | Shrub | Wild, neighborhood | Medicine, trade | Stem, leaf | Stem: decoction and orally taken to treat pulmonary tuberculosis, to promote excretion, purchased by medicinal herb merchants; leaf: mashed and external applied to treat burn and scald | 12 | 0.049 | LFE40181 |
| *Trachycarpus fortunei* (Hook.) H. Wendl. | Zong su | Arecaceae | Tree | Spontaneous species | Food, timber | Flower, bark | Flower: stir-fried or boiled and eaten; bark: to make ropes | 26 | 0.107 | LFE30033 |
| *Asarum splendens* (F. Maek.) C. Y. Cheng & C. S. Yang | Xi xin, ma er mi | Aristolochiaceae | Herb | Wild, neighborhood | Medicine | Root, stem | Decoction and orally taken to rectify Qi and relieve pain, to treat stomachache, headache, lithiasis | 9 | 0.037 | LFE40271 |
| *Agave americana* L. |  | Asparagaceae | Herb | Market | Ornamental | Whole plant | Ornamental | 2 | 0.008 | PLF406018 |
| *Asparagus cochinchinensis* (Lour.) Merr. | Tian dong | Asparagaceae | Herb | Wild | Medicine, trade | Root tuber | Decoction and orally taken to moisten lung and relieve cough, for hemostasis, soaked in wine and orally taken to treat pneumorrhagia; purchased by medicinal herb merchants | 10 | 0.041 | LFE40162 |
| *Asparagus officinalis* L. | Lu sun | Asparagaceae | Herb | Market | Food | Tender stem | Stir-fried and eaten | 1 | 0.004 | PLF404017 |
| *Asparagus setaceus* (Kunth) Jessop |  | Asparagaceae | Liana | Market | Ornamental | Whole plant | Ornamental | 1 | 0.004 | PLF408141 |
| *Aspidistra elatior* Blume | Gan san bian | Asparagaceae | Herb | Wild | Medicine, ornamental | Rhizome, whole plant | Rhizome: soaked in wine or decoction and orally taken to treat traumatic injury; whole plant: ornamental | 3 | 0.012 | PLF408049 |
| *Chlorophytum comosum* (Thunb.) Jacques |  | Asparagaceae | Herb | Market | Ornamental | Whole plant | Ornamental | 6 | 0.025 | PLF408009 |
| *Disporopsis fuscopicta* Hance | Zou gen qi | Asparagaceae | Herb | Wild, market | Medicine, trade | Rhizome | Decoction or soaked in wine and orally taken to treat aching lumbus, traumatic injury, coronary disease, to dispel wind and eliminate dampness; purchased by medicinal herb merchants | 18 | 0.074 | LFE40386 |
| *Diuranthera major* Hemsl. | Xu sen | Asparagaceae | Herb | Wild, neighborhood | Medicine, food, ornamental | Root, whole plant | Root: stewed with meat and eaten to enrich blood reinforce Qi; whole plant: ornamental | 30 | 0.123 | LFE40017 |
| *Hosta plantaginea* (Lam.) Asch. | Yu dan | Asparagaceae | Herb | Spontaneous species | Ornamental | Flower | Ornamental | 4 | 0.016 | PLF404002 |
| *Hosta ventricosa* (Salisb.) Stearn |  | Asparagaceae | Herb | Spontaneous species | Ornamental | Flower | Ornamental | 1 | 0.004 | PLF406007 |
| *Liriope muscari* (Decne.) L. H. Bailey | Da me dong | Asparagaceae | Herb | Spontaneous species | Medicine, food, ornamental, trade | Root tuber | Decoction or soaked in wine and orally taken to eliminate phlegm, to moisten lung and relieve cough, to enrich blood reinforce Qi; stewed with meat and eaten; ornamental; purchased by medicinal herb merchants | 4 | 0.016 | LFE40081 |
| *Ophiopogon japonicus* (L. f.) Ker Gawl. | Me dong | Asparagaceae | Herb | Spontaneous species | Medicine, food, trade | Root tuber | Stewed with meat and eaten to enrich blood reinforce Qi; decoction and orally taken to moisten lung and relieve cough, hemostasis; soaked in wine and orally taken to treat pneumorrhagia; purchased by medicinal herb merchants | 34 | 0.14 | LFE40013 |
| *Peliosanthes macrostegia* Hance | Tie ding pa | Asparagaceae | Herb | Wild | Medicine | Root | Soaked in wine and external applied or orally taken to treat traumatic injury | 1 | 0.004 | PLF403006 |
| *Polygonatum cyrtonema* Hua | Be san qi | Asparagaceae | Herb | Wild, neighborhood | Medicine, trade | Rhizome | Decoction and orally taken to invigorate the kidney, to treat heart disease; purchased by medicinal herb merchants | 2 | 0.008 | LFE40294 |
| *Polygonatum franchetii* Hua | Hong be san qi | Asparagaceae | Herb | Market | Medicine | Rhizome | Decoction and orally taken to invigorate the kidney, to treat heart disease | 1 | 0.004 | LFE40295 |
| *Polygonatum sibiricum* Redouté | Mao jiang | Asparagaceae | Herb | Wild, market | Medicine, food, trade | Rhizome | Roasted or soaked in wine or stewed with meat and eaten; soaked in wine and orally taken to treat aching lumbus, to strengthen muscles and bones, to enrich blood reinforce Qi; purchased by medicinal herb merchants | 124 | 0.51 | LFE30066 |
| *Rohdea chinensis* (Baker) N. Tanaka | Kai kou jian | Asparagaceae | Herb | Wild | Medicine | Rhizome | Decoction and orally taken to relax tendons and activate collaterals | 2 | 0.008 | LFE40282 |
| *Rohdea japonica* (Thunb.) Roth | Bao gu qi | Asparagaceae | Herb | Wild | Medicine, ornamental | Whole plant | Soaked in wine and orally taken to dispel wind and eliminate dampness, to treat traumatic injury and aching lumbus; ornamental | 13 | 0.053 | LFE40296 |
| *Yucca gloriosa* L. |  | Asparagaceae | Shrub | Neighborhood | Ornamental | Whole plant | Ornamental | 4 | 0.016 | PLF408018 |
| *Aloe vera* (L.) Burm. f. | Lu hui | Asphodelaceae | Herb | Market | Ornamental | Whole plant | Ornamental | 2 | 0.008 | PLF310013 |
| *Hemerocallis* citrina Baroni | Jia huang hua | Asphodelaceae | Herb | Neighborhood | Food, ornamental | Flower | Stir-fried and eaten; ornamental | 6 | 0.025 | PLF408060 |
| *Hemerocallis fulva* (L.) L. | Ye huang hua | Asphodelaceae | Herb | Spontaneous species | Food, ornamental | Tender stem and leaf, flower | Stir-fried and eaten; ornamental | 14 | 0.058 | LFE40336 |
| *Achillea millefolium* L. | Yi zi hao | Asteraceae | Herb | Neighborhood | Medicine, ornamental | Whole plant | Mashed and external applied to clear heat and detoxify, to treat snakebite, blister, hemostasis, to relieve pain; ornamental | 7 | 0.029 | LFE40256 |
| *Artemisia argyi* H. Lév. & Vaniot | Ai hao | Asteraceae | Herb | Spontaneous species | Medicine, others | Aboveground | Medicinal bath to dispel wind and eliminate dampness, to treat gynecopathy; as a door hanging to ward off evil spirits on the Dragon Boat Festival | 3 | 0.012 | PLF406016 |
| *Artemisia japonica* Thunb. subf. angustissima (Nakai) Pamp. | Tie gan hao | Asteraceae | Herb | Wild | Medicine | Whole plant | Decoction with eggs and orally taken to treat hepatitis, detumescence | 1 | 0.004 | PLF408024 |
| *Artemisia lactiflora* Wall. ex DC. | Se hao | Asteraceae | Herb | Wild, spontaneous species | Medicine | Leaf | Mashed and external applied to treat blister, mosquito bite, hemostasis | 1 | 0.004 | PLF408044 |
| *Atractylodes macrocephala* Koidz. | Bai suo | Asteraceae | Herb | Market | Medicine, trade | Rhizome | Decoction and orally taken to eliminate phlegm and to tonify spleen; purchased by medicinal herb merchants | 2 | 0.008 | LFE40384 |
| *Chrysanthemum* × *morifolium* (Ramat.) Hemsl. | Ju hua | Asteraceae | Herb | Market, neighborhood | Ornamental | Flower | Ornamental | 26 | 0.107 | PLF311011 |
| *Chrysanthemum indicum* L. | Mao hao | Asteraceae | Herb | Spontaneous species | Medicine, food, ornamental | Flower | Make tea; medicinal bath, to dispel wind and eliminate dampness, to relieve pain; mashed and external applied to treat snakebite; soaked in water and orally taken to improve eyesight; ornamental | 2 | 0.008 | LFE40043 |
| *Cichorium intybus* L. |  | Asteraceae | Herb | Market | Ornamental, forage | Flower, leaf | Flower: ornamental; leaf: forage for fish | 1 | 0.004 | PLF408033 |
| *Cirsium japonicum* Fisch. ex DC. | Da ci ga cai | Asteraceae | Herb | Spontaneous species | Medicine, food | Leaf | Stir-fried or boiled and eaten; decoction and orally taken to promote blood circulation and dispel stasis, mashed and external applied for hemostasis | 1 | 0.004 | LFE40343 |
| *Coreopsis basalis* (A. Dietr.) S. F. Blake |  | Asteraceae | Herb | Market | Ornamental | Flower | Ornamental | 1 | 0.004 | PLF408032 |
| *Cosmos bipinnatus* Cav. |  | Asteraceae | Herb | Market | Ornamental | Flower | Ornamental | 1 | 0.004 | PLF408112 |
| *Dahlia pinnata* Cav. | Sao hua | Asteraceae | Herb | Market, neighborhood | Ornamental | Flower | Ornamental | 19 | 0.078 | PLF406002 |
| *Glebionis coronaria* (L.) Cass. ex Spach | Tong hao | Asteraceae | Herb | Market | Food, trade | Tender stem and leaf | Boiled and eaten; trade it at the market | 122 | 0.502 | PLF403013 |
| *Gynura bicolor* (Roxb. ex Willd.) DC. |  | Asteraceae | Herb | Neighborhood | Food | Tender stem and leaf | Stir-fried and eaten | 3 | 0.012 | PLF406027 |
| *Gynura japonica* (Thunb.) Juel | Xue dang gui | Asteraceae | Herb | Neighborhood | Medicine, ornamental | Whole plant | Decoction and orally taken to treat traumatic injury, gynecopathy, postpartum body pain, headache, hemostasis, to dispel wind and eliminate dampness; ornamental | 14 | 0.058 | LFE40105 |
| *Helianthus annuus* L. | Kui hua | Asteraceae | Herb | Market | Food, ornamental | Seed, flower | Seed: stir-fried and eaten; flower: ornamental | 3 | 0.012 | PLF408146 |
| *Helianthus tuberosus* L. | Yang jiang | Asteraceae | Herb | Self-preservation | Food, ornamental | Tuber | As pickle and eaten; ornamental | 26 | 0.107 | LFE40470 |
| *Lactuca sativa* var. *angustata* Irish ex Bremer | Wo sun | Asteraceae | Herb | Market | Food, forage, trade | Stem, leaf | Stem: stir-fried or boiled and eaten, forage for pigs, trade it at the market; leaf: forage for pigs | 206 | 0.848 | PLF408109 |
| *Lactuca sativa* var. *asparagina* L.H.Bailey ex Holub | You mai cai | Asteraceae | Herb | Market | Food, forage | Leaf | Stir-fried and eaten; forage for pigs and poultry | 33 | 0.136 | PLF311016 |
| *Lactuca sativa* var. *ramosa* Hort. | Sen cai | Asteraceae | Herb | Market | Food, forage, trade | Leaf | Stir-fried and eaten; forage for pigs and poultry; trade it at the market | 48 | 0.198 | PLF310012 |
| *Ligularia hodgsonii* Hook. | Qian cui da bu si | Asteraceae | Herb | Wild | Medicine, ornamental | Root | Soaked in wine and external applied to dispel wind and eliminate dampness, to treat traumatic injury | 2 | 0.008 | LFE40237 |
| *Pseudognaphalium affine* (D. Don) Anderb. | Sui qiao | Asteraceae | Herb | Spontaneous species | Food, forage | Tender stem and leaf | Made cakes with glutinous rice flour and steamed and eaten; forage for pigs | 12 | 0.049 | LFE30013 |
| *Senecio scandens* Buch.-Ham. ex D. Don | Jiu lin guang | Asteraceae | Herb | Spontaneous species | Medicine, ornamental | Whole plant | Medicinal bath to dispel wind and eliminate dampness, to relieve pain, to treat blister; mashed and external applied to treat skin disease; soaked in water to boil eggs, roll eggs around the eyes to improve eyesight; ornamental | 1 | 0.004 | LFE40045 |
| *Sigesbeckia orientalis* L. | Xi xian cao | Asteraceae | Herb | Spontaneous species | Medicine | Whole plant | Decoction and orally taken or external applied to dispel wind and eliminate dampness, to treat snakebite | 1 | 0.004 | PLF408028 |
| *Smallanthus sonchifolius* (Poeppig) H. Rob. | Xue lian guo | Asteraceae | Herb | Market | Food | Tuber | Eaten directly | 11 | 0.045 | PLF406015 |
| *Solidago decurrens* Lour. | Cai fu | Asteraceae | Herb | Wild | Medicine | Whole plant | Decoction and orally taken to treat hepatitis and lithiasis | 1 | 0.004 | LFE40111 |
| *Tagetes erecta* L. |  | Asteraceae | Herb | Market | Ornamental | Flower | Ornamental | 5 | 0.021 | PLF408062 |
| *Taraxacum mongolicum* Hand.-Mazz. | Pu gong yin | Asteraceae | Herb | Spontaneous species | Medicine, food | Aboveground, whole plant | Aboveground: stir-fried or made cold dishes and eaten; whole plant: decoction and orally taken to treat pulmonary nodule, coronary disease, angina pectoris, gynecopathy, gout, blister; soaked in wine and external applied to treat traumatic injury | 4 | 0.016 | LFE40197 |
| *Zinnia elegans* Jacq. |  | Asteraceae | Herb | Market | Ornamental | Flower | Ornamental | 9 | 0.037 | PLF408008 |
| *Impatiens balsamina* L. | Zi ga hua | Balsaminaceae | Herb | Spontaneous species | Medicine, ornamental | Seed, flower | Seed: decoction and orally taken to treat lithiasis; Flower: ornamental | 72 | 0.296 | LFE30057 |
| *Basella alba* L. | Mu er cai | Basellaceae | Herb | Self-preservation | Food, ornamental | Leaf, whole plant | Leaf: stir-fried or make soup and eaten; whole plant: ornamental | 12 | 0.049 | PLF408074 |
| *Begonia grandis* Dryand. |  | Begoniaceae | Herb | Market | Ornamental | Whole plant | Ornamental | 2 | 0.008 | PLF408069 |
| *Begonia pedatifida* H. Lév. | Yi kou xue | Begoniaceae | Herb | Wild | Medicine | Leaf, root | Leaf: mashed and external applied for hemostasis; root: mashed and external applied, or soaked in wine and orally taken, to treat traumatic injury | 1 | 0.004 | PLF311002 |
| *Dysosma majoensis* (Gagnep.) M. Hiroe | Hong ba guo lian | Berberidaceae | Herb | Wild | Medicine | Leaf, root | Soaked in wine or decoction, orally taken or external applied, to treat traumatic injury, blister, snakebite, to dispel wind and eliminate dampness | 2 | 0.008 | PLF404007 |
| *Dysosma versipellis* (Hance) M. Cheng | Ba guo lian | Berberidacseae | Herb | Wild | Medicine | Leaf, root | Soaked in wine or decoction, orally taken or external applied, to treat traumatic injury, blister, snakebite, to dispel wind and eliminate dampness | 1 | 0.004 | PLF404011 |
| *Epimedium sagittatum* (Siebold & Zucc.) Maxim. | Tie da chu, yin yang huo | Berberidaceae | Herb | Wild | Medicine, trade | Whole plant | Medicinal bath or orally taken to dispel wind and eliminate dampness, to relieve pain, to treat gynecopathy, to invigorate the kidney; purchased by medicinal herb merchants | 9 | 0.037 | LFE40236 |
| *Mahonia bealei* (Fortune) Carr. | Ci huang lian | Berberidaceae | Shrub | Wild | Medicine, trade | Root, stem | Decoction and orally taken, or soaked in wine and orally taken or external applied, to clear heat, to disperse swelling and relieve pain, for hemostasis, as antidiarrheal, to treat traumatic injury, mouth sores, pneumonia; purchased by medicinal herb merchants | 9 | 0.037 | LFE40410 |
| *Mahonia fortunei* (Lindl.) Fedde | Ci huang lian | Berberidaceae | Shrub | Wild | Medicine, trade | Root, stem | Decoction and orally taken, or soaked in wine and orally taken or external applied, to clear heat, to disperse swelling and relieve pain, for hemostasis, as antidiarrheal, to treat traumatic injury, mouth sores, pneumonia; purchased by medicinal herb merchants | 3 | 0.012 | LFE30003 |
| *Nandina domestica* Thunb. | Nan din qi | Berberidaceae | Shrub | Market | Medicine, ornamental | Whole plant | Decoction or soaked in wine and orally taken to treat traumatic injury; ornamental | 6 | 0.025 | PLF408118 |
| *Campsis grandiflora* (Thunb.) Schum. | Ten luo hua | Bignoniaceae | Liana | Market | Ornamental | Flower | Ornamental | 7 | 0.029 | PLF408081 |
| *Brassica juncea* (L.) Czern. | Jie cai | Brassicaceae | Herb | Market | Food, forage | Leaf | Stir-fried or as pickle and eaten; forage for pigs | 60 | 0.247 | PLF408057 |
| *Brassica juncea* var. *napiformis* (Pailleux & Bois) Kitam. | Da tou cai | Brassicaceae | Herb | Market | Food, forage, trade | Root tuber | As pickle and eaten; forage for pigs; trade it at the market | 148 | 0.609 | PLF311018 |
| *Brassica juncea* var. *tumida* Tsen & S. H. Lee | Za cai | Brassicaceae | Herb | Market | Food, forage | Petiole, stem | Stir-fried or as pickle and eaten; forage for pigs | 113 | 0.465 | PLF311015 |
| *Brassica rapa* L. | Bian luo bu | Brassicaceae | Herb | Market | Food, forage | Root tuber, whole plant | Root tuber: boiled or as pickle and eaten; whole plant: forage for pigs and poultry | 70 | 0.288 | PLF311017 |
| *Brassica rapa* var. *chinensis* (L.) Kitam. | Qin cai | Brassicaceae | Herb | Market | Food, forage, trade | Leaf | Stir-fried or boiled and eaten; forage for pigs and poultry; trade it at the market | 218 | 0.897 | PLF310004 |
| *Brassica rapa* var. *oleifera* DC. | Cai tai | Brassicaceae | Herb | Market | Food, forage, trade | Tender stem and leaf, flower | Stir-fried or boiled and eaten; forage for pigs and poultry; trade it at the market | 120 | 0.494 | PLF311014 |
| *Raphanus sativus* L. | Luo bu | Brassicaceae | Herb | Market | Food, forage, trade | Root, whole plant | Root: boiled and eaten, trade it at the market; whole plant: forage for pigs | 222 | 0.914 | PLF310002 |
| *Brassica juncea* var. *gemmifera* Lee & Lin | Er cai | Brassicaceae | Herb | Market | Food, trade | Stem | Stir-fried and eaten; trade it at the market | 84 | 0.346 | PLF310003 |
| *Brassica oleracea* var. *capitata* L. | Qiu be | Brassicaceae | Herb | Market | Food, forage | Leaf | Stir-fried or boiled and eaten; forage for pigs and poultry | 116 | 0.477 | PLF311013 |
| *Brassica rapa* var. *glabra* Regel | Be cai | Brassicaceae | Herb | Market, self-preservation | Food, forage, trade | Leaf | Stir-fried or boiled and eaten; forage for pigs and poultry; trade it at the market | 214 | 0.881 | PLF311012 |
| *Buxus bodinieri* H. Lév. | Qian lian ai | Buxaceae | Shrub | Neighborhood | Ornamental | Whole plant | Ornamental | 25 | 0.103 | PLF408002 |
| *Sarcococca hookeriana* var. *digyna* Franch. | Dou gen | Buxaceae | Shrub | Wild | Medicine | Root | Decoction and orally taken to treat stomachache | 1 | 0.004 | PLF408026 |
| *Echinopsis tubiflora* (Pfeiff.) Zucc. ex A. Dietr. | Xian en qiu | Cactaceae | Herb | Market | Ornamental | Whole plant | Ornamental | 1 | 0.004 | PLF408099 |
| *Opuntia dillenii* (Ker Gawl.) Haw. | Xian en zang | Cactaceae | Shrub | Market | Medicine, ornamental | Whole plant | Mashed and external applied to treat lymphadenitis and blister | 11 | 0.045 | PLF408042 |
| *Selenicereus undatus* (Haw.) D.R.Hunt | Huo long guo | Cactaceae | Shrub | Market | Ornamental | Whole plant | Ornamental | 1 | 0.004 | PLF408068 |
| *Codonopsis javanica* subsp. *japonica* (Makino) Lammers | Ye dang sen | Campanulaceae | Liana | Wild, spontaneous species | Medicine, food | Root | Stewed with meat and eaten to enrich blood reinforce Qi, to strengthen muscles and bones | 7 | 0.029 | LFE30044 |
| *Platycodon grandiflorus* (Jacq.) A. DC. |  | Campanulaceae | Herb | Market | Ornamental | Flower | Ornamental | 1 | 0.004 | PLF408080 |
| *Trema cannabina* var. *dielsiana* (Hand.-Mazz.) C. J. Chen | Ai sa zi su | Cannabaceae | Shrub | Spontaneous species | Ornamental | Whole plant | Ornamental | 1 | 0.004 | LFE40465 |
| *Canna indica* L. | Mei en jiao | Cannaceae | Herb | Neighborhood | Ornamental | Flower | Ornamental | 16 | 0.066 | PLF408061 |
| *Lonicera japonica* Thunb. | Jin yin hua | Caprifoliaceae | Liana | Spontaneous species | Medicine, food, ornamental, trade | Whole plant, flower | Whole plant: medicinal bath to treat blister; decoction and orally taken to treat cough and cold, throatache, pulmonary nodule, bronchitis, as antidiarrheal; flower: make tea, ornamental, purchased by medicinal herb merchants | 2 | 0.008 | LFE40140 |
| *Weigela florida* (Bunge) A. DC. |  | Caprifoliaceae | Shrub | Market | Ornamental | Flower | Ornamental | 1 | 0.004 | LFE40308 |
| Dianthus chinensis L. |  | Caryophyllaceae | Herb | Market | Ornamental | Flower | Ornamental | 8 | 0.033 | PLF408004 |
| *Euonymus alatus* (Thunb.) Siebold | Suo luo su | Celastraceae | Shrub | Market | Medicine, ornamental | Stem, bark | Stem: decoction and orally taken to treat heart disease; bark: decoction and orally taken to treat fever; ornamental | 7 | 0.029 | PLF406011 |
| *Euonymus japonicus* Thunb. | Qian lian ai | Celastraceae | Shrub | Neighborhood | Ornamental | Whole plant | Ornamental | 15 | 0.062 | PLF408108 |
| *Chloranthus henryi* Hemsl. | Si kuai wa | Chloranthaceae | Herb | Wild | Medicine | Whole plant | Stewed with meat and eaten to enrich blood reinforce Qi; boiled with eggs and eaten to calm; decoction and orally taken to treat toothache, postpartum body pain, hepatitis; mashed and external applied to treat traumatic injury | 1 | 0.004 | LFE40333 |
| *Sarcandra glabra* (Thunb.) Nakai | Guan yin xiang | Chloranthaceae | Shrub | Wild, market | Medicine, trade | Whole plant | Decoction and orally taken to clear heat and detoxify; purchased by medicinal herb merchants | 1 | 0.004 | LFE40240 |
| *Tarenaya hassleriana* (Chodat) Iltis |  | Cleomaceae | Herb | Market | Ornamental | Flower | Ornamental | 2 | 0.008 | PLF408079 |
| *Disporum longistylum* (H. Lév. & Vaniot) H. Hara | Be long xu | Colchicaceae | Herb | Spontaneous species | Medicine, food | Rhizome | Stewed with meat and eaten to enrich blood reinforce Qi | 4 | 0.016 | LFE40001 |
| *Pollia japonica* Thunb. | Ye yang huo | Commelinaceae | Herb | Spontaneous species | Medicine | Whole plant | Decoction, and orally taken to treat traumatic injury and stomachache | 1 | 0.004 | LFE40332 |
| *Tradescantia ohiensis* Raf. |  | Commelinaceae | Herb | Market | Ornamental | Whole plant | Ornamental | 1 | 0.004 | PLF408087 |
| *Tradescantia pallida* (Rose) D. R. Hunt |  | Commelinaceae | Herb | Market | Ornamental | Whole plant | Ornamental | 8 | 0.033 | LFE30017 |
| *Tradescantia zebrina* Bosse |  | Commelinaceae | Herb | Market, neighborhood | Ornamental | Whole plant | Ornamental | 4 | 0.016 | PLF408147 |
| *Ipomoea aquatica* Forssk. | Kong xin cai | Convolvulaceae | Herb | Market | Food, forage, trade | Stem, leaf | Stir-fried and eaten; forage for pigs; trade it at the market | 40 | 0.165 | PLF406028 |
| *Ipomoea batatas* (L.) Lam. | sao | Convolvulaceae | Liana | Self-preservation | Food, forage, trade | Tender stem and leaf, root tuber | Tender stem and leaf: stir-fried and eaten, forage for pigs, trade it at the market; root tuber: steamed or boiled and eaten, forage for pigs, trade it at the market | 116 | 0.477 | PLF408078 |
| *Ipomoea quamoclit* L. |  | Convolvulaceae | Herb | Market | Ornamental | Flower | Ornamental | 1 | 0.004 | PLF408140 |
| *Alangium chinense* (Lour.) Harms | Be jin tiao | Cornaceae | Shrub | Spontaneous species | Medicine | Root | Soaked in wine and orally taken to treat traumatic injury, to dispel wind and eliminate dampness; decoction and orally taken to treat irregular menses | 6 | 0.025 | PLF408027 |
| *Cornus capitata* Wall. | Li ba zi | Cornaceae | Tree | Spontaneous species | Food, ornamental | Fruit, flower | Fruit: eaten directly; flower: ornamental | 2 | 0.008 | LFE40253 |
| *Cornus macrophylla* Wall. | Deng tai su | Cornaceae | Tree | Spontaneous species | Ornamental, timber | Stem | Ornamental; firewood | 14 | 0.058 | PLF408096 |
| *Aeonium arboreum* (L.) Webb et Berthel. | Duo ou | Crassulaceae | Shrub | Market | Ornamental | Whole plant | Ornamental | 2 | 0.008 | PLF408138 |
| *Crassula arborescens* (Mill.) Willd. | Duo ou | Crassulaceae | Shrub | Market | Ornamental | Whole plant | Ornamental | 1 | 0.004 | PLF310014 |
| *Echeveria secunda* Booth ex Lindl. | Duo ou | Crassulaceae | Herb | Market | Ornamental | Whole plant | Ornamental | 3 | 0.012 | PLF408135 |
| *Graptopetalum paraguayense* (N.E.Br.) E.Walther | Duo ou | Crassulaceae | Shrub | Market | Ornamental | Whole plant | Ornamental | 17 | 0.07 | PLF408064 |
| *Hylotelephium erythrostictum* (Miq.) H. Ohba | Fu dou qi | Crassulaceae | Herb | Spontaneous species | Food | Aboveground | Stir-fried or made cold dishes and eaten | 2 | 0.008 | LFE40362 |
| *Sedum emarginatum* Migo | Ma ci han | Crassulaceae | Herb | Spontaneous species | Food | Aboveground | Stir-fried or made cold dishes and eaten | 1 | 0.004 | LFE30049 |
| *Sedum sediforme* Pau | Duo ou | Crassulaceae | Herb | Market | Ornamental | Whole plant | Ornamental | 1 | 0.004 | PLF408145 |
| *Phedimus aizoon* (L.) 't Hart | Da fu si | Crassulaceae | Herb | Wild, neighborhood | Medicine, ornamental | Whole plant | Soaked in wine and orally taken or external applied to mashed and external applied, to treat traumatic injury and sprain, hemostasis; ornamental | 9 | 0.037 | LFE30098 |
| *Sedum hispanicum* L. |  | Crassulaceae | Herb | Market | Ornamental | Whole plant | Ornamental | 4 | 0.016 | PLF408134 |
| *Sedum sarmentosum* Bunge | Gou ya pan | Crassulaceae | Herb | Spontaneous species | Medicine, food | Whole plant, aboveground | Whole plant: mashed and external applied to treat dog bite, traumatic injury; aboveground: stir-fried or made cold dishes and eaten | 3 | 0.012 | LFE40231 |
| *Benincasa hispida* (Thunb.) Cogn. | Dong gua | Cucurbitaceae | Herb | Market, self-preservation | Food, forage, trade | Fruit | Stir-fried or boiled and eaten; forage for pigs and poultry; trade it at the market | 145 | 0.597 | PLF408065 |
| *Citrullus lanatus* (Thunb.) Matsum. & Nakai | Xi gua | Cucurbitaceae | Herb | Market | Food, trade | Fruit | Eaten directly; trade it at the market | 32 | 0.132 | PLF408123 |
| *Cucumis melo* L. | Xiang gua | Cucurbitaceae | Herb | Market | Food, trade | Fruit | Eaten directly; trade it at the market | 32 | 0.132 | PLF406026 |
| *Cucumis sativus* L. | Huang gua | Cucurbitaceae | Herb | Self-preservation | Food, trade | Fruit | Stir-fried or boiled and eaten; trade it at the market | 187 | 0.77 | PLF408037 |
| *Cucurbita moschata* (Duchesne ex Lam.) Duchesne ex Poir. | Nan gua | Cucurbitaceae | Herb | Market, self-preservation | Medicine, food, forage, trade | Tender stem and leaf, fruit, root | Root: mashed and external applied to treat lymphadenitis; tender stem and leaf: stir-fried; fruit: stir-fried or boiled and eaten, forage for pigs and poultry; trade it at the market | 214 | 0.881 | PLF408107 |
| *Cucurbita pepo* L. |  | Cucurbitaceae | Herb | Market | Food, trade | Fruit | Stir-fried and eaten; trade it at the market | 93 | 0.383 | PLF408129 |
| *Hemsleya chinensis* Cogn. ex F. B. Forbes & Hemsl. | Bei wei lian, san wu gui | Cucurbitaceae | Herb | Wild | Medicine, trade | Root tuber | Soaked in wine and external applied to treat traumatic injury | 2 | 0.008 | LFE40381 |
| *Lagenaria siceraria* (Molina) Standl. | Fu lou | Cucurbitaceae | Herb | Market | Food, ornamental | Fruit | Stir-fried or boiled and eaten; trade it at the market | 4 | 0.016 | PLF408019 |
| *Luffa aegyptiaca* Mill. | Si gua | Cucurbitaceae | Liana | Self-preservation | Food, trade | Fruit | Stir-fried or boiled and eaten; trade it at the market | 151 | 0.621 | PLF408040 |
| *Momordica charantia* L. | Ku gua | Cucurbitaceae | Herb | Self-preservation | Food, trade | Fruit | Stir-fried and eaten; trade it at the market | 198 | 0.815 | PLF408039 |
| *Sechium edule* (Jacq.) Sw. | Fu er gua | Cucurbitaceae | Liana | Market | Food, forage | Fruit | Stir-fried and eaten, forage for pigs and poultry | 1 | 0.004 | PLF408139 |
| *Trichosanthes kirilowii* Maxim. | Gou si gua | Cucurbitaceae | Liana | Self-preservation | Medicine, trade | Fruit | Decoction and orally taken to treat coronary disease, angina pectoris, child's nocturia, to moisten lung and relieve cough; purchased by medicinal herb merchants | 6 | 0.025 | LFE40273 |
| *Carex siderosticta* Hance |  | Cyperaceae | Herb | Wild | Ornamental | Whole plant | Ornamental | 1 | 0.004 | LFE40434 |
| *Cyperus involucratus* Rottb. |  | Cyperaceae | Herb | Market | Ornamental | Whole plant | Ornamental | 1 | 0.004 | PLF408128 |
| *Dioscorea alata* L. | Jiu ban sao | Dioscoreaceae | Liana | Self-preservation | Food | Tuber | Steamed or roasted and eaten | 2 | 0.008 | LFE40289 |
| *Dioscorea bulbifera* L. | Mao quan tou | Dioscoreaceae | Liana | Spontaneous species | Food | Tuber | Steamed or roasted and eaten | 1 | 0.004 | LFE40412 |
| *Diospyros cathayensis* Steward | Jin dan zi | Ebenaceae | Tree | Wild | Ornamental, trade | Whole plant | As bonsai for ornamental and sale | 28 | 0.115 | LFE30092 |
| *Diospyros kaki* Thunb. | Si zi | Ebenaceae | Tree | Market | Food | Fruit | Eaten directly | 43 | 0.177 | LFE40409 |
| *Elaeagnus henryi* Warb. ex Diels | Yang nai nai | Elaeagnaceae | Shrub | Wild | Food, ornamental | Fruit, stem | Stem: ground into powder and orally taken to treat bronchitis; fruit: eaten directly; ornamental | 2 | 0.008 | LFE30020 |
| *Elaeagnus magna* (Servett.) Rehder | Fan quan zi | Elaeagnaceae | Shrub | Wild | Medicine, food, ornamental | Fruit, root | Root: decoction and orally taken as antiabortifacient; fruit: eaten directly; ornamental | 7 | 0.029 | LFE40395 |
| *Elaeagnus pungens* Thunb. | Niu nai nai | Elaeagnaceae | Shrub | Wild | Medicine, food, ornamental | Fruit, root | Root: decoction, orally taken, to treat pulmonary tuberculosis, as antidiarrheal; fruit: eaten directly; ornamental | 13 | 0.053 | LFE30019 |
| *Rhododendron simsii* Planch. | Yan san hong | Ericaceae | Shrub | Wild | Food, ornamental | Flower | Eaten directly; ornamental | 20 | 0.082 | LFE40303 |
| *Eucommia ulmoides* Oliv. | Si mian | Eucommiaceae | Tree | Wild, spontaneous species | Medicine, ornamental, trade | Bark, tender leaf | Bark: soaked in wine and orally taken to treat aching lumbus, traumatic injury, gynecopathy, to invigoratie the kidney, to dispel wind and eliminate dampness, soaked in water and held in mouth to treat toothache; tender leaf: mashed and external applied for hemostasis; ornamental; purchased by medicinal herb merchants | 11 | 0.045 | LFE40386 |
| *Euphorbia humifusa* Willd. ex Schltdl. | Pu di ma | Euphorbiaceae | Herb | Spontaneous species | Medicine | Whole plant | Mashed and soaked in rice water and orally taken to treat dysentery and mouth sores | 1 | 0.004 | PLF311004 |
| *Euphorbia pekinensis* Rupr. | Gua ding ban | Euphorbiaceae | Herb | Wild | Medicine | Root | Decoction and orally taken to treat traumatic injury | 1 | 0.004 | PLF408015 |
| *Mallotus philippensis* (Lamarck) Müll. Arg. |  | Euphorbiaceae | Tree | Spontaneous species | Ornamental | Whole plant | Ornamental | 1 | 0.004 | LFE40429 |
| *Ricinus communis* L. | Bi ma | Euphorbiaceae | Herb | Self-preservation | Medicine, trade | Seed | Mashed and external applied to treat blister; purchased by medicinal herb merchants | 4 | 0.016 | PLF408066 |
| *Triadica sebifera* (L.) Small | Mu you zi su | Euphorbiaceae | Tree | Spontaneous species | Ornamental, timber | Whole plant | Ornamental; firewood | 6 | 0.025 | PLF408117 |
| *Vernicia fordii* (Hemsl.) Airy Shaw | Tong zi su | Euphorbiaceae | Tree | Market | Medicine, ornamental, trade, others | Seed, flower, root | Seed: extract oil and external applied to treat blister and child's fever, as antidiarrheal, for hemostasis, to relieve pain, extract oil and make into paint and lamp oil for sale; flower: ornamental; root: soaked in wine and external applied to treat traumatic injury, to disperse swelling and relieve pain | 4 | 0.016 | LFE40452 |
| *Arachis hypogaea* L. | Hua sen | Fabaceae | Herb | Self-preservation | Food, trade | Seed | Eaten directly or boiled and eaten; trade it at the market | 6 | 0.025 | PLF408130 |
| *Canavalia gladiata* (Jacq.) DC. | Dao ba dou | Fabaceae | Herb | Market, self-preservation | Food | Fruit | Stir-fried and eaten | 3 | 0.012 | PLF408084 |
| *Caragana sinica* (Buc'hoz) Rehder | Yang quo hua | Fabaceae | Shrub | Wild | Food, ornamental | Flower, root | Flower: stir-fried or make soup and eaten, ornamental; root: decoction and orally taken to treat nephritis | 16 | 0.066 | LFE40160 |
| *Cercis chinensis* Bunge |  | Fabaceae | Shrub | Market | Ornamental | Whole plant | Ornamental | 23 | 0.095 | PLF408012 |
| *Cheniella tenuiflora* (Watt ex C. B. Clarke) R. Clark & Mackinder | Zu yao zi ten | Fabaceae | Liana | Wild | Medicine, ornamental | Whole plant | Soaked in wine or decoction or stewed with pig kidney and eaten to treat aching lumbus, for hemostasis; ornamental | 2 | 0.008 | LFE40328 |
| *Glycine max* (L.) Merr. | Huang dou | Fabaceae | Herb | Self-preservation | Food, trade | Seed | Stir-fried or boiled or made tofu and eaten; trade it at the market | 39 | 0.16 | PLF311010 |
| *Lablab purpureus* (L.) Sweet | Wo mei dou | Fabaceae | Liana | Market | Food, trade | Fruit | Stir-fried or boiled and eaten; trade it at the market | 20 | 0.082 | PLF408132 |
| *Pachyrhizus erosus* (L.) Urb. | Liang su | Fabaceae | Liana | Market | Food, trade | Root tuber | Eaten directly or stir-fried and eaten; trade it at the market | 16 | 0.066 | PLF408031 |
| *Phaseolus vulgaris* L. | Si ji dou | Fabaceae | Herb | Market | Food, trade | Fruit | Stir-fried and eaten; trade it at the market | 201 | 0.827 | PLF408102 |
| *Pisum sativum* L. | Wan dou | Fabaceae | Herb | Market, self-preservation | Food | Tender stem and leaf, fruit, seed | Tender stem and leaf: stir-fried and eaten; tender fruit: stir-fried and eaten; seed: boiled and eaten | 112 | 0.461 | PLF403001 |
| *Robinia pseudoacacia* L. | Ci huai | Fabaceae | Tree | Spontaneous species | Food, ornamental | Flower | Make soup and eaten; ornamental | 9 | 0.037 | LFE30027 |
| *Senna occidentalis* (L.) Link |  | Fabaceae | Shrub | Self-preservation | Medicine | Stem, leaf | Mashed and external applied to treat snakebite | 1 | 0.004 | PLF406013 |
| *Senna tora* (L.) Roxb. |  | Fabaceae | Herb | Market | Medicine | Seed | Decoction and orally taken to improve eyesight | 3 | 0.012 | PLF408076 |
| *Styphnolobium japonicum* (L.) Schott | Huai su | Fabaceae | Tree | Spontaneous species | Ornamental, timber | Stem | Ornamental; firewood | 2 | 0.008 | PLF408127 |
| *Vigna radiata* (L.) R. Wilczek | Lu dou | Fabaceae | Herb | Self-preservation | Food, trade | Seed | Boiled and eaten; trade it at the market | 2 | 0.008 | PLF408137 |
| *Vigna unguiculata* subsp. *sesquipedalis* (L.) Verdc. | Gang dou | Fabaceae | Herb | Market | Food, trade | Fruit | Stir-fried or as pickle and eaten; trade it at the market | 209 | 0.86 | PLF408045 |
| *Castanea mollissima* Blume | Ban li | Fagaceae | Tree | Neighborhood, self-preservation | Food, timber, trade | Seed, stem | Seed: eaten directly or boiled and eaten, trade it at the market; stem: firewood | 38 | 0.156 | LFE30100 |
| *Castanopsis eyrei* (Champ. ex Benth.) Tutcher | Si li | Fagaceae | Tree | Spontaneous species | Food, timber | Seed, stem | Seed: eaten directly or boiled and eaten; stem: firewood | 2 | 0.008 | LFE30025 |
| *Castanopsis tibetana* Hance | Hou li | Fagaceae | Tree | Spontaneous species | Food, timber | Seed, stem | Stem: construction material, made roof beams for stilt buildings; seed: eaten directly or boiled and eaten | 1 | 0.004 | LFE30052 |
| *Quercus glauca* Thunb. | Qing gang | Fagaceae | Tree | Spontaneous species | Ornamental, timber | Stem | Ornamental; firewood | 5 | 0.021 | LFE30059 |
| *Pelargonium hortorum* L. H. Bailey |  | Geraniaceae | Herb | Market | Ornamental | Flower | Ornamental | 2 | 0.008 | PLF408100 |
| *Hemiboea subcapitata* C. B. Clarke | Ai que zi | Gesneriaceae | Herb | Spontaneous species | Ornamental | Whole plant, flower | Whole plant: mashed and external applied to treat burn and scald; flower: ornamental | 1 | 0.004 | LFE40447 |
| *Distylium racemosum* Siebold & Zucc. |  | Hamamelidaceae | Shrub | Market | Ornamental | Whole plant | Ornamental | 1 | 0.004 | PLF408142 |
| *Loropetalum chinense* var. *rubrum* Yieh |  | Hamamelidaceae | Shrub | Market | Ornamental | Whole plant | Ornamental | 7 | 0.029 | PLF408119 |
| *Dichroa febrifuga* Lour. | Chang san | Hydrangeaceae | Shrub | Spontaneous species | Medicine, ornamental | Root | Decoction and orally taken to treat cough and cold, malaria; soaked in wine and orally taken or external applied to treat traumatic injury; ornamental | 1 | 0.004 | LFE40143 |
| *Hydrangea macrophylla* (Thunb.) Ser. | Xiu qiu | Hydrangeaceae | Shrub | Market | Ornamental | Flower | Ornamental | 5 | 0.021 | PLF408005 |
| *Nothapodytes pittosporoides* (Oliv.) Sleumer | Lai ke ma su | Icacinaceae | Tree | Wild | Medicine, ornamental | Root, whole plant | Root: mashed and external applied to treat hemorrhoid; Whole plant: ornamental | 2 | 0.008 | LFE40310 |
| *Belamcanda chinensis* (L.) Redouté | Zong sou san fu | Iridaceae | Herb | Wild, market | Medicine, ornamental, trade | Rhizome, flower | Rhizome: decoction and orally taken to treat throatache and cough and cold, purchased by medicinal herb merchants; flower: ornamental | 4 | 0.016 | PLF408010 |
| *Gladiolus gandavensis* Van Houtte | San pan qi | Iridaceae | Herb | Neighborhood | Medicine, ornamental | Corm, flower | Corm: soaked in wine and orally taken or external applied to treat traumatic injury; flower: ornamental | 21 | 0.086 | LFE30015 |
| *Iris confusa* Sealy | Sou san fu | Iridaceae | Herb | Wild, neighborhood | Medicine, ornamental | Rhizome, flower | Rhizome: mashed and external applied, or soaked in wine and orally taken to treat traumatic injury; flower: ornamental | 3 | 0.012 | LFE40361 |
| *Iris tectorum* Maxim. | Sou san fu | Iridaceae | Herb | Market | Medicine, ornamental | Rhizome, flower | Rhizome: mashed and external applied, or soaked in wine and orally taken to treat traumatic injury; flower: ornamental | 2 | 0.008 | PLF404009 |
| *Iris wilsonii* C. H. Wright | Kai kou jian | Iridaceae | Herb | Wild | Medicine | Rhizome | Decoction and orally taken to treat throatache and pulmonary tuberculosis | 2 | 0.008 | LFE40245 |
| *Juglans regia* L. | He tao | Juglandaceae | Tree | Market | Food, timber | Seed, stem | Seed: eaten directly; stem: firewood | 24 | 0.099 | LFE30058 |
| *Agastache rugosa* (Fisch. & C. A. Mey.) Kuntze | Huo xiang cao | Lamiaceae | Herb | Neighborhood | Medicine, food, ornamental | Leaf | As spice; mashed with rice water and orally taken to clear heat and detoxify, as antidiarrheal; ornamental | 8 | 0.033 | LFE40366 |
| *Callicarpa giraldii* Hesse ex Rehder |  | Lamiaceae | Shrub | Wild | Medicine, ornamental | Fruit, leaf | Mashed with tea oil and external applied to treat blister | 1 | 0.004 | LFE40438 |
| *Clerodendrum bungei* Steud. | Cou mou dan | Lamiaceae | Shrub | Spontaneous species | Medicine, ornamental | Root, flower | Root: decoction and orally taken, stewed with meat and eaten, made cakes with glutinous rice flour and eaten, to enrich blood reinforce Qi; flower: ornamental | 9 | 0.037 | LFE40079 |
| *Glechoma longituba* (Nakai) Kupr. | Tong qian cao | Lamiaceae | Herb | Spontaneous species | Medicine | Whole plant | Mashed and external applied to treat blister; decoction and orally taken to treat lithiasis | 6 | 0.025 | LFE40257 |
| *Leonurus japonicus* Houtt. | Yi mi cao | Lamiaceae | Herb | Spontaneous species | Medicine | Whole plant | Decoction or soaked in wine and orally taken to treat gynecopathy, traumatic injury, colpomycosis, irregular menses, to promote blood circulation and dispel stasis | 3 | 0.012 | LFE40292 |
| *Lycopus lucidus* Turcz. ex Benth. | Di luo si | Lamiaceae | Herb | Market | Medicine, food | Rhizome, whole plant | Rhizome: stir-fried or made cold dishes and eaten; whole plant: decoction and orally taken to treat gynecopathy | 1 | 0.004 | LFE40012 |
| *Mentha canadensis* L. | Bo huo | Lamiaceae | Herb | Wild, neighborhood | Medicine, food, ornamental, others | Whole plant | Decoction and orally taken to treat cough and cold, blister, skin disease, hepatitis; mashed and external applied to improve eyesight; medicinal bath to ward off evil spirits on the Dragon Boat Festival; ornamental | 6 | 0.025 | LFE40338 |
| *Nepeta cataria* L. | Xiao jin gai | Lamiaceae | Herb | Spontaneous species | Medicine | Whole plant | Decoction and orally taken to treat cough and cold, blister, hepatitis; medicinal bath to dispel wind and eliminate dampness, to relieve pain | 4 | 0.016 | LFE30099 |
| *Perilla frutescens* (L.) Britton | Zi su | Lamiaceae | Herb | Self-preservation | Medicine, food | Leaf | As spice for cooking fish to remove fishy smell; decoction and orally taken to treat cough and cold, coronary disease, angina pectoris | 79 | 0.325 | LFE40350 |
| *Perilla frutescens* var. *crispa* (Thunb.) Hand.-Mazz. | Zi su | Lamiaceae | Herb | Self-preservation | Medicine, food | Root, leaf | Root: decoction and orally taken to treat gynecopathy; leaf: as spice | 10 | 0.041 | LFE40312 |
| *Premna microphylla* Turcz. | Ban ji pao | Lamiaceae | Shrub | Spontaneous species | Food | Leaf, whole plant | Leaf: made tofu and eaten; mashed and external applied for hemostasis; whole plant: medicinal bath to dispel wind and eliminate dampness | 1 | 0.004 | LFE40376 |
| *Prunella vulgaris* L. | Hong kuo cao | Lamiaceae | Herb | Spontaneous species | Medicine, food | Aboveground | Make tea; decoction and orally taken to dispel wind and eliminate dampness, to treat cough and cold, pulmonary tuberculosis, promote diuresis, blister | 1 | 0.004 | LFE40109 |
| *Salvia cavaleriei* H. Lév. | Fan bei hong | Lamiaceae | Herb | Spontaneous species | Medicine | Whole plant | Mashed with rice water and external applied to treat mouth sores; decoction and orally taken to relieve cough | 1 | 0.004 | LFE40226 |
| *Salvia miltiorrhiza* Bunge | Dan sen | Lamiaceae | Herb | Market | Medicine | Root | Decoction and orally taken to treat irregular menses | 1 | 0.004 | PLF408048 |
| *Salvia splendens* Ker Gawl. |  | Lamiaceae | Herb | Market | Ornamental | Flower | Ornamental | 8 | 0.033 | PLF408029 |
| *Scutellaria barbata* D. Don | Ban bian lian | Lamiaceae | Herb | Spontaneous species | Medicine | Whole plant | Mashed and external applied to treat snakebite, blister, inflammation; decoction and orally taken, or medicinal bath, to dispel wind and eliminate dampness, to relieve pain, to treat irregular menses, gynecopathy | 1 | 0.004 | PLF408051 |
| *Akebia trifoliata* (Thunb.) Koidz. | Ba yue gua | Lardizabalaceae | Liana | Wild, market | Food | Fruit | Eaten directly | 3 | 0.012 | LFE40394 |
| *Cinnamomum pauciflorum* Chun ex Hung T. Chang | Xiang gui zi | Lauraceae | Tree | Wild | Food | Bark | As spice | 1 | 0.004 | LFE30038 |
| *Litsea elongata* (Wall. ex Ness) Benth. & Hook. f. |  | Lauraceae | Tree | Market | Ornamental | Whole plant | Ornamental | 1 | 0.004 | PLF408043 |
| *Litsea mollis* Hemsl. | Mu jiang zi | Lauraceae | Tree | Wild | Food, trade | Fruit | As spice or made cold dishes and eaten; trade it at the market | 7 | 0.029 | LFE40020 |
| *Machilus nanmu* (Oliv.) Hemsl. | Zu ye lan | Lauraceae | Tree | Spontaneous species | Ornamental | Whole plant | Ornamental | 1 | 0.004 | PLF408072 |
| *Phoebe zhennan* S. K. Lee & F. N. Wei | Jin si lan mu | Lauraceae | Tree | Wild, spontaneous species | Ornamental, timber | Whole plant | Ornamental; furniture material | 24 | 0.099 | PLF408063 |
| *Fritillaria monantha* Migo | Bei mu | Liliaceae | Herb | Wild | Medicine, trade | Bulb | Soaked in wine or decoction and orally taken to moisten lung and relieve cough; purchased by medicinal herb merchants | 3 | 0.012 | PLF404005 |
| *Lilium brownii* F. E. Br. ex Miellez | Ye be huo | Liliaceae | Herb | Spontaneous species | Food, ornamental | Bulb, flower | Bulb: roasted or steamed and eaten; flower: ornamental | 1 | 0.004 | LFE40431 |
| *Lilium brownii* var. *viridulum* Baker | Be huo | Liliaceae | Herb | Market, neighborhood | Ornamental | Bulb, flower | Bulb: decoction and orally taken to treat stomachache, to relieve cough; flower: ornamental | 9 | 0.037 | PLF406022 |
| *Torenia fournieri* Linden ex E. Fourn. | Tong hai cao | Linderniaceae | Herb | Spontaneous species | Medicine | Whole plant | Decoction and orally taken to treat dyspepsia, to calm | 1 | 0.004 | PLF408023 |
| *Cuphea hookeriana* Walp. |  | Lythraceae | Shrub | Market | Ornamental | Flower | Ornamental | 4 | 0.016 | PLF408083 |
| *Lagerstroemia indica* L. | Zi wei | Lythraceae | Shrub | Market | Ornamental | Whole plant | Ornamental | 98 | 0.403 | PLF408122 |
| *Punica granatum* L. | Si liu | Lythraceae | Shrub | Market | Medicine, food, ornamental | Root, fruit, flower | Root: decoction and orally taken as antidiarrheal; fruit: eaten directly, ornamental; flower: ornamental | 7 | 0.029 | PLF408125 |
| *Houpoea officinalis* (Rehder & E. H. Wilson) N. H. Xia & C. Y. Wu |  | Magnoliaceae | Tree | Market | Medicine, trade | Bark | Decoction and orally taken to treat stomachache, cough and cold, coronary disease, lithiasis, nephritis; purchased by medicinal herb merchants | 4 | 0.016 | PLF404001 |
| *Yulania* × *soulangeana* (Soul.-Bod.) D. L. Fu | Yu lan hua | Magnoliaceae | Tree | Market | Ornamental | Flower | Ornamental | 7 | 0.029 | PLF403009 |
| *Abelmoschus esculentus* (L.) Moench | Qiu kui | Malvaceae | Herb | Market | Food | Fruit | Stir-fried and eaten | 2 | 0.008 | PLF408136 |
| *Abelmoschus manihot* (L.) Medik. | Zui xian tao | Malvaceae | Herb | Market | Ornamental | Flower | Ornamental | 4 | 0.016 | PLF408077 |
| *Alcea rosea* L. |  | Malvaceae | Herb | Market | Ornamental | Flower | Ornamental | 5 | 0.021 | PLF408131 |
| *Firmiana simplex* (L.) W. Wight | Tong ma su | Malvaceae | Tree | Spontaneous species | Ornamental, timber | Whole plant | Ornamental; firewood | 2 | 0.008 | PLF408114 |
| *Hibiscus mutabilis* L. | Fu yong hua | Malvaceae | Shrub | Spontaneous species | Ornamental | Flower | Ornamental | 1 | 0.004 | PLF310015 |
| *Hibiscus syriacus* L. | Mu jin hua | Malvaceae | Shrub | Market | Ornamental | Flower | Ornamental | 15 | 0.062 | PLF408007 |
| *Paris cronquistii* (Takht.) H. Li | Qi ye yi zi hua, tie deng tai | Melanthiaceae | Herb | Wild | Medicine, trade | Rhizome | Soaked in wine and orally taken or external applied, or mashed and external applied, to treat snakebite, aching lumbus, detumescence, blister, traumatic injury, detumescence, stomachache; purchased by medicinal herb merchants | 9 | 0.037 | PLF404008 |
| *Paris delavayi* Franch. | Qi ye yi zi hua, tie deng tai | Melanthiaceae | Herb | Wild | Medicine, trade | Rhizome | Soaked in wine and orally taken or external applied, or mashed and external applied to treat snakebite, aching lumbus, detumescence, blister, traumatic injury, detumescence, stomachache; purchased by medicinal herb merchants | 15 | 0.062 | LFE40130 |
| *Paris fargesii* Franch. | Qi ye yi zi hua, tie deng tai | Melanthiaceae | Herb | Wild | Medicine, trade | Rhizome | Soaked in wine and orally taken or external applied, or mashed and external applied to treat snakebite, aching lumbus, detumescence, blister, traumatic injury, detumescence, stomachache; purchased by medicinal herb merchants | 5 | 0.021 | PLF404006 |
| *Paris thibetica* Franch. | Qi ye yi zi hua, tie deng tai | Melanthiaceae | Herb | Wild | Medicine, trade | Rhizome | Soaked in wine and orally taken or external applied, or mashed and external applied to treat snakebite, aching lumbus, detumescence, blister, traumatic injury, detumescence, stomachache; purchased by medicinal herb merchants | 7 | 0.029 | PLF403007 |
| *Melia azedarach* L. | Ku lian | Meliaceae | Tree | Spontaneous species | Medicine, ornamental, timber | Bark, stem | Bark: medicinal bath to treat skin disease; stem: firewood; ornamental | 7 | 0.029 | PLF404013 |
| *Toona sinensis* (Juss.) Roem. | Xiang cun | Meliaceae | Tree | Spontaneous species | Medicine, food, timber, trade | Tender stem and leaf, stem, bark | Tender stem and leaf: stir-fried or made cold dishes and eaten, trade it at the market; stem: firewood, construction material; bark: decoction and external applied to treat eczema | 17 | 0.07 | LFE40170 |
| *Stephania cephalantha* Hayata | San wu gui | Menispermaceae | Liana | Wild | Medicine | Root | Soaked in wine and external applied to treat traumatic injury, detumescence | 2 | 0.008 | PLF408003 |
| *Tinospora sagittata* (Oliv.) Gagnep. | Di ku dan | Menispermaceae | Liana | Wild | Medicine, trade | Root tuber | Decoction and orally taken to clear heat and detoxify, to treat lithiasis, gout, stomachache; purchased by medicinal herb merchants | 4 | 0.016 | LFE40225 |
| *Ficus carica* L. | Wu hua guo | Moraceae | Shrub | Market | Food, ornamental | Fruit | Eaten directly | 5 | 0.021 | PLF408091 |
| *Ficus tikoua* Bureau | Di pi pa | Moraceae | Liana | Spontaneous species | Medicine, food | Fruit, whole plant, tender stem and leaf | Fruit: eaten directly; whole plant: decoction and orally taken to treat cough and cold, eliminate phlegm; tender stem and leaf: eaten directly to treat gastroenteritis | 1 | 0.004 | LFE40038 |
| *Morus alba* L. | Sang su | Moraceae | Shrub | Market | Forage | Leaf | Forage for silkworms | 8 | 0.033 | LFE40098 |
| *Musa basjoo* Siebold & Zucc. ex Iinuma | Ba jiao | Musaceae | Herb | Spontaneous species | Medicine, food, ornamental, forage | Leaf, flower, tender stem and leaf | Leaf: packaging materials for cakes, forage for pigs; flower: stewed with meat and eaten to treat heart disease; tender stem and leaf: boiled and eaten; ornamental | 26 | 0.107 | LFE30104 |
| *Morella rubra* Lour. | Yang mei | Myricaceae | Tree | Market | Food, ornamental, trade | Fruit | Eaten directly or soaked in wine and eaten, ornamental, trade it at the market | 31 | 0.128 | LFE40252 |
| *Nelumbo nucifera* Gaertn. | Huo hua | Nelumbonaceae | Herb | Market | Food, ornamental | Rhizome, flower | Rhizome: stir-fried or boiled and eaten; flower: ornamental | 3 | 0.012 | PLF408126 |
| *Bougainvillea spectabilis* Willd. |  | Nyctaginaceae | Shrub | Market | Ornamental | Flower | Ornamental | 1 | 0.004 | PLF408143 |
| *Mirabilis jalapa* L. |  | Nyctaginaceae | Herb | Spontaneous species | Ornamental | Flower | Ornamental | 4 | 0.016 | PLF406023 |
| *Nymphaea tetragona* Georgi |  | Nymphaeaceae | Herb | Market | Ornamental | Flower | Ornamental | 2 | 0.008 | PLF408070 |
| *Fraxinus hubeiensis* S.Z.Qu, C.B.Shang & P.L.Su |  | Oleaceae | Tree | Market | Ornamental | Whole plant | Ornamental | 1 | 0.004 | PLF408105 |
| *Jasminum nudiflorum* Lindl. | Yin cun hua | Oleaceae | Shrub | Market | Ornamental | Flower | Ornamental | 1 | 0.004 | PLF403012 |
| *Ligustrum lucidum* W. T. Aiton | Be la su | Oleaceae | Tree | Spontaneous species | Ornamental, timber | Whole plant | Ornamental; firewood | 2 | 0.008 | PLF408058 |
| *Ligustrum quihoui* Carrière | Xiao be la su | Oleaceae | Shrub | Market | Ornamental | Whole plant | Ornamental | 1 | 0.004 | PLF408034 |
| *Osmanthus fragrans* (Thunb.) Lour. | Gui hua su | Oleaceae | Shrub | Market, neighborhood | Ornamental | Flower | Ornamental | 117 | 0.481 | PLF408052 |
| *Bletilla striata* (Thunb. ex A. Murray) Rchb. f. | Be ji | Orchidaceae | Herb | Wild, market | Medicine, trade | Tuber | Decoction and orally taken to treat cough and cold、gout; mashed and external applied for hemostasis; purchased by medicinal herb merchants | 6 | 0.025 | PLF406009 |
| *Changnienia amoena* S. S. Chien | Bin zu zi, mao ci gu | Orchidaceae | Herb | Wild | Medicine, trade | Pseudobulb | Decoction or soaked in wine and orally taken to treat cough and cold, pulmonary nodule; purchased by medicinal herb merchants | 1 | 0.004 | PLF403008 |
| *Cremastra appendiculata* (D. Don) Makino | Bin zu zi, mao ci gu | Orchidaceae | Herb | Wild | Medicine, trade | Pseudobulb | Decoction or soaked in wine and orally taken to treat cough and cold, pulmonary nodule; purchased by medicinal herb merchants | 3 | 0.012 | LFE40227 |
| *Cymbidium ensifolium* (L.) Sw. | Lan cao | Orchidaceae | Herb | Wild | Ornamental | Flower | Ornamental | 4 | 0.016 | PLF408021 |
| *Cymbidium faberi* Rolfe | Lan cao | Orchidaceae | Herb | Wild | Ornamental | Flower | Ornamental | 7 | 0.029 | PLF311009 |
| *Cymbidium floribundum* Lindl. | Lan cao | Orchidaceae | Herb | Wild | Ornamental | Flower | Ornamental | 2 | 0.008 | PLF404019 |
| *Cymbidium goeringii* (Rchb. f.) Rchb. F. | Lan cao | Orchidaceae | Herb | Wild | Ornamental | Flower | Ornamental | 5 | 0.021 | PLF404018 |
| *Cymbidium kanran* Makino | Lan cao | Orchidaceae | Herb | Wild | Ornamental | Flower | Ornamental | 9 | 0.037 | PLF310008 |
| *Dendrobium wilsonii* Rolfe | Huang cao | Orchidaceae | Herb | Wild | Medicine, ornamental, trade | Whole plant | Decoction and orally taken to treat heart disease, stomachache; ornamental; purchased by medicinal herb merchants | 5 | 0.021 | LFE40413 |
| *Liparis nervosa* (Thunb. ex A. Murray) Lindl. | Jian xue qin | Orchidaceae | Herb | Wild | Medicine, ornamental | Whole plant | Decoction and orally taken to moisten lung and relieve cough; soaked in wine and orally taken or external applied to treat traumatic injury; ornamental | 1 | 0.004 | LFE40415 |
| *Oxalis corniculata* L. | Lao wa xuan | Oxalidaceae | Herb | Spontaneous species | Medicine | Whole plant | Mashed and external applied to treat blister, traumatic injury; mashed and orally taken as antidiarrheal; decoction and orally taken to treat gynecopathy, aching lumbus; soaked in wine and orally taken or external applied to treat traumatic injury, to relieve pain | 1 | 0.004 | LFE40180 |
| *Paeonia* × *suffruticosa* Andrews | Mou dan | Paeoniaceae | Shrub | Market | Ornamental | Flower | Ornamental | 3 | 0.012 | PLF406012 |
| *Paeonia lactiflora* Pall. | Suo yo | Paeoniaceae | Herb | Market | Medicine, ornamental | Root, flower | Root: decoction and orally taken to treat stomachache, gynecopathy, to promote blood circulation and dispel stasis, throatache, colpomycosis, traumatic injury; flower: ornamental | 11 | 0.045 | PLF408006 |
| *Paulownia fortunei* (Seem.) Hemsl. | Pao tong | Paulowniaceae | Tree | Spontaneous species | Ornamental, timber | Whole plant | Ornamental; firewood | 1 | 0.004 | PLF408093 |
| *Phyllanthus urinaria* L. | Ye xia zu | Phyllanthaceae | Herb | Wild | Medicine | Whole plant | Decoction and orally taken to clear heat and detoxify | 2 | 0.008 | PLF408016 |
| *Plantago asiatica* L. | Ke ma cao | Plantaginaceae | Herb | Spontaneous species | Medicine, food | Whole plant, aboveground | Whole plant: decoction and orally taken to treat cough and cold; aboveground: stir-fried or boiled and eaten | 3 | 0.012 | LFE40107 |
| *Veronicastrum stenostachyum* subsp. *plukenetii* (T. Yamaz.) D. Y. Hong | Diao yu gan | Plantaginaceae | Herb | Spontaneous species | Medicine | Leaf, whole plant | Leaf: mashed with tea oil and external applied to treat blister; whole plant: decoction and orally taken to disperse swelling and relieve pain, to expel parasite | 1 | 0.004 | PLF408025 |
| *Ceratostigma plumbaginoides* Bunge |  | Plumbaginaceae | Herb | Market | Ornamental | Flower | Ornamental | 1 | 0.004 | PLF408089 |
| *Bambusa* *emeiensis* L. C. Chia & H. L. Fung | Peng zou, ci zou | Poaceae | Bamboo | Neighborhood | Food, ornamental, timber | Stem | Tender stem: boiled or stir-fried and eaten; old stem: woven material; ornamental | 28 | 0.115 | LFE40399 |
| *Coix lacryma-jobi* L. | Niao zu zi | Poaceae | Herb | Neighborhood | Medicine | Seed | Decoction and orally taken to treat lithiasis, to promote diuresis | 5 | 0.021 | PLF408116 |
| *Indocalamus tessellatus* (Munro) P. C. Keng | Zong ba ye | Poaceae | Bamboo | Spontaneous species | Food, trade | Leaf | Soaked in wine and eaten; packaging materials for cakes; trade it at the market | 31 | 0.128 | LFE30040 |
| *Phyllostachys edulis* (Carrière) J. Houz. | Lan zou | Poaceae | Bamboo | Neighborhood, self-preservation | Food, ornamental, timber, trade | Stem | Tender stem: boiled or stir-fried and eaten; old stem: woven material, construction material, firewood; ornamental | 27 | 0.111 | LFE30029 |
| *Phyllostachys heteroclada* Oliv. | Sui zou | Poaceae | Herb | Neighborhood | Food, ornamental, timber | Stem | Tender stem: boiled or stir-fried and eaten; old stem: woven material; ornamental | 1 | 0.004 | LFE30031 |
| *Phyllostachys nidularia* Munro | Be zou | Poaceae | Bamboo | Neighborhood | Food, ornamental, timber | Stem | Tender stem: boiled or stir-fried and eaten; old stem: woven material; ornamental | 1 | 0.004 | LFE30034 |
| *Phyllostachys nigra* var. *henonis* (Mitford) Stapf ex Rendle | Jin zou | Poaceae | Bamboo | Neighborhood | Food, ornamental, timber | Stem | Tender stem: boiled or stir-fried and eaten; old stem: woven material; ornamental | 5 | 0.021 | LFE40097 |
| *Pleioblastus maculatus* (McClure) C. D. Chu & C. S. Chao | Ku zou | Poaceae | Bamboo | Neighborhood | Food, ornamental, timber | Stem | Tender stem: boiled or stir-fried and eaten; old stem: woven material; ornamental | 1 | 0.004 | LFE40391 |
| *Saccharum officinarum* L. | Gan za | Poaceae | Herb | Self-preservation | Food, forage | Rhizome, leaf | Rhizome: eaten directly; leaf: forage for livestock | 1 | 0.004 | PLF408067 |
| *Sorghum bicolor* (L.) Moench | Gao liang | Poaceae | Herb | Market | Forage | Seed, stem, leaf | Forage for livestock | 2 | 0.008 | PLF408046 |
| *Zea mays* L. | Bao gu | Poaceae | Herb | Market | Food, forage, trade | Fruit, whole plant | Fruit: boiled or stir-fried or steamed and eaten, trade it at the market; whole plant: forage for livestock | 150 | 0.617 | PLF408013 |
| *Zizania latifolia* (Griseb.) Turcz. ex Stapf | Gao sun | Poaceae | Herb | Market | Food | Tender stem | Stir-fried and eaten | 3 | 0.012 | PLF408020 |
| *Fagopyrum dibotrys* (D. Don) Hara | Qiao zi qi | Polygonaceae | Herb | Spontaneous species | Medicine, forage | Root | Decoction and orally taken to dispel wind and eliminate dampness, to treat traumatic injury, dysentery, as antidiarrheal; forage for pigs and poultry | 9 | 0.037 | LFE40077 |
| *Persicaria runcinata* var. *sinensis* (Hemsl.) Bo Li | Hong ce lan | Polygonaceae | Herb | Wild, spontaneous species | Medicine | Leaf | Boiled or stir-fried with eggs and eaten to treat headache, gynecopathy, pneumonia | 6 | 0.025 | LFE40234 |
| *Pleuropterus multiflorus* (Thunb.) Nakai | Ye jiao ten | Polygonaceae | Herb | Spontaneous species | Medicine | Root tuber | Decoction and orally taken to treat gastroenteritis, insomnia and dreaminess, dysentery, to enrich blood reinforce Qi | 5 | 0.021 | LFE40177 |
| *Reynoutria japonica* Houtt. | Suan tan gen | Polygonaceae | Herb | Neighborhood | Medicine, food, trade | Tender stem, rhizome | Tender stem: eaten directly or made cold dishes and eaten; rhizome: medicinal bath to dispel wind and eliminate dampness, to relieve lumbago and leg pain, boiled into paste and external applied to treat burn and scald, decoction and orally taken to treat hepatitis, epilepsy; purchased by medicinal herb merchants | 10 | 0.041 | LFE40184 |
| *Rumex japonicus* Houtt. | Da huang | Polygonaceae | Herb | Spontaneous species | Medicine | Root | Soaked in wine and orally taken or external applied to treat traumatic injury; decoction and orally taken to treat headache, bronchitis, to promote excretion; medicinal bath to treat blister | 5 | 0.021 | LFE40070 |
| *Rumex nepalensis* Spreng. | Liu da huang | Polygonaceae | Herb | Spontaneous species | Medicine | Root | Soaked in wine and orally taken or external applied to treat traumatic injury; decoction and orally taken to treat headache, bronchitis, to promote excretion; medicinal bath to treat blister | 3 | 0.012 | LFE30016 |
| *Rumex obtusifolius* L. | Xue si da huang | Polygonaceae | Herb | Wild | Medicine | Root | Soaked in wine and orally taken or external applied to treat traumatic injury; decoction and orally taken to treat headache, bronchitis, to promote excretion; medicinal bath to treat blister | 2 | 0.008 | PLF403004 |
| *Portulaca grandiflora* Hook. | Tai yang hua | Portulacaceae | Herb | Neighborhood | Ornamental | Flower | Ornamental | 2 | 0.008 | PLF408090 |
| *Ardisia crenata* Sims | Kai hou jian | Primulaceae | Shrub | Wild, market | Medicine, ornamental | Whole plant | Decoction and orally taken to treat throatache, pharyngitis; ornamental | 9 | 0.037 | LFE40093 |
| *Lysimachia christinae* Hance | Guo lu huang | Primulaceae | Herb | Neighborhood, spontaneous species | Medicine | Whole plant | Decoction and orally taken to treat urethral calculus; mashed and external applied to treat blister, as antiphlogistic | 2 | 0.008 | LFE40102 |
| *Lysimachia paridiformis* Franch. | Si kuai wa | Primulaceae | Herb | Wild | Medicine, food, trade | Whole plant | Stewed with meat and eaten to enrich blood reinforce Qi; boiled with eggs and eaten to calm; decoction and orally taken to treat toothache, postpartum body pain, hepatitis; mashed and external applied to treat traumatic injury; trade it at the market | 6 | 0.025 | LFE40158 |
| *Aconitum carmichaelii* Debeaux | Wu dou | Ranunculaceae | Herb | Wild | Medicine | Root tuber | Mashed with wine and external applied to treat snakebite, detumescence, blister; wrap and orally taken to treat traumatic injury, aching lumbus | 2 | 0.008 | LFE40291 |
| *Clematis finetiana* H. Lév. & Vaniot | Wei lin xian | Ranunculaceae | Liana | Wild | Medicine, trade | Root | Decoction and orally taken to dispel wind and eliminate dampness, relax tendons and activate collaterals, lithiasis; purchased by medicinal herb merchants | 1 | 0.004 | PLF404020 |
| *Coptis chinensis* Franch. | Huang lian | Ranunculaceae | Herb | Market | Medicine, trade | Rhizome | Decoction and orally taken to treat hepatitis, to clear heat; purchased by medicinal herb merchants | 4 | 0.016 | LFE40218 |
| *Hovenia acerba* Lindl. | Guai zao | Rhamnaceae | Tree | Spontaneous species | Medicine, food, timber, trade | Fruit, fruit stalk, stem | Eaten directly or soaked in wine and eaten; soaked in wine and orally taken or external applied to relax tendons and activate collaterals, to dispel wind and eliminate dampness、bronchitis; stem: furniture material; trade it at the market | 32 | 0.132 | LFE40034 |
| *Ziziphus jujuba* (L.) Lam. | Zao | Rhamnaceae | Shrub | Neighborhood | Food, ornamental | Fruit | Eaten directly; ornamental | 12 | 0.049 | PLF408011 |
| *Chaenomeles cathayensis* (Hemsl.) C. K. Schneid. | Mu gua | Rosaceae | Shrub | Neighborhood | Medicine, food, ornamental, trade | Fruit, flower | Fruit: soaked in wine and orally taken to dispel wind and eliminate dampness, purchased by medicinal herb merchants; flower: ornamental | 10 | 0.041 | LFE40065 |
| *Eriobotrya japonica* (Thunb.) Lindl. | Pi pa | Rosaceae | Tree | Neighborhood | Medicine, food, ornamental | Fruit, flower, leaf | Fruit: eaten directly; flower, leaf: decoction and orally taken to treat cough and cold, pulmonary nodule, gout | 81 | 0.333 | LFE40249 |
| *Fragaria* × *ananassa* (Weston) Duchesne ex Rozier | Cao mei | Rosaceae | Herb | Market | Food | Fruit | Eaten directly | 7 | 0.029 | PLF406025 |
| *Kerria japonica* (L.) DC. |  | Rosaceae | Shrub | Wild, spontaneous species | Ornamental | Flower | Ornamental | 5 | 0.021 | PLF403003 |
| *Malus spectabilis* (Aiton) Borkh. | Hai tang | Rosaceae | Tree | Market | Ornamental | Flower | Ornamental | 1 | 0.004 | PLF403011 |
| *Photinia serratifolia* (Desf.) Kalkman |  | Rosaceae | Shrub | Market | Ornamental | Whole plant | Ornamental | 2 | 0.008 | PLF408115 |
| *Prunus mume* Siebold & Zucc. | Mei hua | Rosaceae | Tree | Market | Ornamental | Flower | Ornamental | 5 | 0.021 | PLF403010 |
| *Prunus persica* (L.) Batsch | Tao | Rosaceae | Tree | Market | Food, ornamental | Fruit, flower | Fruit: eaten directly; flower: ornamental | 79 | 0.325 | PLF408106 |
| *Prunus pseudocerasus* Lindl. | En tao | Rosaceae | Tree | Market | Food, ornamental | Flower, fruit | Flower: ornamental; fruit: eaten directly | 20 | 0.082 | LFE40255 |
| *Prunus salicina* Lindl. | Mei li | Rosaceae | Tree | Market | Food, ornamental | Fruit, flower | Fruit: eaten directly; flower: ornamental | 76 | 0.313 | PLF408086 |
| *Pyracantha fortuneana* (Maxim.) H. L. Li | Jiu bei liang | Rosaceae | Shrub | Wild | Medicine, food, ornamental | Fruit, seed, leaf | Fruit: eaten directly or soaked in wine and eaten; seed: medicinal bath to treat gynecopathy, stanch bleeding; leaf: mashed and external applied for hemostasis; ornamental | 10 | 0.041 | LFE30009 |
| *Pyrus pyrifolia* (Burm. F.) Nakai | Li | Rosaceae | Tree | Neighborhood | Food, ornamental | Fruit, flower | Fruit: eaten directly; flower: ornamental | 56 | 0.23 | PLF406017 |
| *Rosa chinensis* Jacq. | Yue yue hong | Rosaceae | Shrub | Market, neighborhood | Medicine, ornamental | Root, flower | Decoction and orally taken to treat gynecopathy, irregular menses; ornamental | 54 | 0.222 | PLF404004 |
| *Rosa multiflora* Thunb. | Yue yue hong | Rosaceae | Shrub | Market | Medicine, ornamental | Root, flower | Decoction and orally taken to treat gynecopathy, irregular menses; ornamental | 12 | 0.049 | PLF406001 |
| *Rosa roxburghii* f. *normalis* Rehd.et Wils. | Ci guo guo | Rosaceae | Shrub | Wild | Medicine, food, ornamental | Fruit, root, flower | Fruit: eaten directly or soaked in wine and eaten; root: medicinal bath to treat gynecopathy; flower: ornamental | 2 | 0.008 | LFE40347 |
| *Rubus rosifolius* Sm. | Kong tong pao | Rosaceae | Shrub | Spontaneous species | Medicine, food | Fruit, tender stem and leaf | Fruit: eaten directly; tender stem and leaf: orally taken to treat gastroenteritis | 1 | 0.004 | LFE30018 |
| *Rubus wallichianus* Wight & Arn. | Hou zi pao | Rosaceae | Shrub | Wild | Medicine, food, ornamental | Fruit, root | Root: decoction and orally taken to treat gastroenteritis; fruit: eaten directly; ornamental | 3 | 0.012 | LFE40392 |
| *Gardenia jasminoides* J. Ellis | Zi zi hua | Rubiaceae | Shrub | Market, neighborhood | Medicine, ornamental | Flower, fruit | Flower: ornamental; fruit: decoction and orally taken to treat hepatitis, to clear heat and detoxify | 23 | 0.095 | PLF406004 |
| *Serissa japonica* (Thunb.) Thunb. | Qian lian ai | Rubiaceae | Shrub | Spontaneous species | Ornamental | Whole plant | Ornamental | 2 | 0.008 | PLF406024 |
| *Citrus* × *aurantium* Siebold & Zucc. ex Engl. | Gou gan zi | Rutaceae | Tree | Spontaneous species | Others | Whole plant | As a fence planted on the edge of the homegarden | 14 | 0.058 | PLF406019 |
| *Citrus maxima* (Burm.) Merr. | You zi | Rutaceae | Tree | Market, neighborhood | Medicine, food, ornamental | Fruit | Boil fruit and rolled it away on the body to treat cough and cold, headache; eaten directly; trade it at the market | 107 | 0.44 | PLF310005 |
| *Citrus reticulata* Blanco | Ju zi | Rutaceae | Tree | Market | Food | Fruit | Eaten directly | 41 | 0.169 | PLF408111 |
| *Citrus sinensis* (L.) Osbeck | Cen zi | Rutaceae | Tree | Market | Food | Fruit | Eaten directly | 4 | 0.016 | PLF310007 |
| *Phellodendron amurense* Rupr. | Huang be | Rutaceae | Tree | Market | Medicine, trade | Bark | Decoction and orally taken to clear heat and detoxify | 18 | 0.074 | LFE40215 |
| *Tetradium ruticarpum* (A. Juss.) T. G. Hartley | Su you zi | Rutaceae | Tree | Wild, spontaneous species | Medicine, trade | Flower, fruit | Soaked in wine or decoction and orally taken to treat stomachache, dyspepsia, to rectify Qi and relieve pain; purchased by medicinal herb merchants | 10 | 0.041 | LFE40196 |
| *Zanthoxylum armatum* DC. | Ye hua jiao | Rutaceae | Tree | Wild | Food | Fruit | As spice | 1 | 0.004 | LFE40427 |
| *Zanthoxylum bungeanum* Maxim. | Hua jiao | Rutaceae | Tree | Market | Food | Fruit, tender leaf, root | Fruit: as spice, soaked in water and held in mouth to treat toothache, medicinal bath to relax tendons and activate collaterals, to dispel wind and eliminate dampness; tender leaf: stir-fried or boiled and eaten; root: decoction and held in mouth to treat toothache | 41 | 0.169 | LFE40428 |
| *Idesia polycarpa* Maxim. | Sui dong gua | Salicaceae | Tree | Spontaneous species | Ornamental, timber | Fruit, stem | Fruit: ornamental; stem: firewood | 1 | 0.004 | PLF310006 |
| *Acer palmatum* Thunb. | Hong feng | Sapindaceae | Tree | Market | Ornamental | Whole plant | Ornamental | 1 | 0.004 | PLF404014 |
| *Koelreuteria bipinnata* Franch. | Deng long su | Sapindaceae | Tree | Spontaneous species | Ornamental, timber | Whole plant, stem | Stem: firewood; whole plant: ornamental | 2 | 0.008 | PLF408104 |
| *Houttuynia cordata* Thunb. | Ce er gen | Saururaceae | Herb | Wild, spontaneous species | Medicine, food, trade | Whole plant, rhizome | Rhizome: made cold dishes and eaten; whole plant: decoction and orally taken to treat cough and cold, inflammation, to clear heat, mashed with wine and external applied for hemostasis, mashed and external applied to treat bee sting, medicinal bath to treat blister; purchased by medicinal herb merchants | 67 | 0.276 | LFE40039 |
| *Heuchera micrantha* Douglas ex Lindl. |  | Saxifragaceae | Herb | Market | Ornamental | Whole plant | Ornamental | 1 | 0.004 | PLF408144 |
| *Saxifraga stolonifera* Meerb. | Fu er cao | Saxifragaceae | Herb | Neighborhood, spontaneous species | Medicine, ornamental | Whole plant | Mashed and squeezed out the juice and drip it into the ear to treat tympanitis; ornamental | 7 | 0.029 | LFE40075 |
| *Illicium henryi* Diels | Ba guo xiang | Schisandraceae | Tree | Wild | Food, ornamental | Fruit | As spice; ornamental | 7 | 0.029 | LFE40095 |
| *Schisandra henryi* C. B. Clarke | Cen tuo | Schisandraceae | Liana | Wild | Medicine, food | Fruit | Eaten directly; decoction and orally taken to treat traumatic injury, to promote blood circulation and dispel stasis, to treat cough and cold, stomachache | 1 | 0.004 | LFE40369 |
| *Dipsacus asper* Wall. | Dang sen | Scrophulariaceae | Herb | Wild | Medicine | Root, leaf | Mashed and external applied to treat traumatic injury | 1 | 0.004 | PLF311001 |
| *Alkekengi officinarum* Moench | Tian pao zi | Solanaceae | Herb | Neighborhood | Medicine, ornamental | Fruit | Mashed and soaked in tea oil and external applied to treat blister; ornamental | 5 | 0.021 | LFE40374 |
| *Brugmansia arborea* (L.) Lagerh. | Lao yang hua | Solanaceae | Shrub | Neighborhood | Ornamental | Flower | Ornamental | 3 | 0.012 | PLF310001 |
| *Capsicum annuum* L. | La zi | Solanaceae | Herb | Market, self-preservation | Food, trade | Fruit | Stir-fried and eaten or as spice; trade it at the market | 209 | 0.86 | PLF408036 |
| *Lycium chinense* Mill. | Gou qi | Solanaceae | Shrub | Neighborhood | Food, ornamental | Fruit | Soaked in wine and eaten; ornamental | 11 | 0.045 | LFE40348 |
| *Solanum lycopersicum* L. | Jiang guo | Solanaceae | Herb | Market | Food, trade | Fruit | Stir-fried or made cold dishes and eaten; trade it at the market | 165 | 0.679 | PLF408041 |
| *Solanum lyratum* Thunb. | Mao er duo | Solanaceae | Liana | Spontaneous species | Medicine | Fruit, whole plant | Fruit: mashed and held in mouth to treat toothache; whole plant: medicinal bath to treat vaginitis | 1 | 0.004 | LFE40049 |
| *Solanum melongena* L. | Que zi | Solanaceae | Herb | Market | Medicine, food, trade | Flower, fruit | Flower: decoction and orally as acyeterion; fruit: stir-fried and eaten, trade it at the market | 188 | 0.774 | PLF408038 |
| *Solanum pseudocapsicum* L. |  | Solanaceae | Shrub | Market | Ornamental | Fruit | Ornamental | 8 | 0.033 | PLF408133 |
| *Solanum tuberosum* L. | Yang yu | Solanaceae | Herb | Self-preservation | Food, forage, trade | Tuber | Stir-fried or boiled and eaten; forage for pigs; trade it at the market | 104 | 0.428 | PLF406014 |
| *Talinum paniculatum* (Jacq.) Gaertn. | Yang sen | Talinaceae | Herb | Spontaneous species | Medicine, food, ornamental | Root, leaf | Root: stewed with meat and eaten to enrich blood reinforce Qi; leaf: stir-fried or boiled and eaten; ornamental | 9 | 0.037 | LFE40403 |
| *Camellia japonica* L. | Ca hua | Theaceae | Tree | Market | Ornamental | Flower | Ornamental | 8 | 0.033 | PLF408120 |
| *Camellia oleifera* Abel | You ca | Theaceae | Shrub | Market | Medicine, food, timber, trade | Seed, gall, leaf, bark, stem | Seed: extract oil and eaten, external applied to treat blister, trade; gall: eaten directly; leaf: mashed and external applied, hemostasis; bark: mashed and external applied for hemostasis; stem: firewood | 33 | 0.136 | LFE40110 |
| *Camellia sinensis* (L.) Kuntze | Ca | Theaceae | Shrub | Self-preservation | Food, timber, trade | Leaf, stem | Tender leaf: make tea and eaten, trade it at the market; stem: firewood | 28 | 0.115 | PLF408103 |
| *Stewartia sinensis* Rehder & E. H. Wilson | Ma lin guang | Theaceae | Tree | Wild | Ornamental, timber | Whole plant | Ornamental; firewood | 1 | 0.004 | PLF403002 |
| *Edgeworthia chrysantha* Lindl. | Meng hua | Thymelaeaceae | Shrub | Market | Ornamental | Flower | Ornamental | 10 | 0.041 | PLF406021 |
| *Torricellia angulata* var. *intermedia* (Harms) Hu | Lan li ba | Torricelliaceae | Tree | Wild | Medicine | Root | Soaked in wine or decoction, orally taken or external applied, to dispel wind and eliminate dampness, to treat traumatic injury, to promote blood circulation and dispel stasis, to disperse swelling and relieve pain | 6 | 0.025 | LFE40198 |
| *Boehmeria nivea* (L.) Gaudich. | Ye ma | Urticaceae | Shrub | Spontaneous species | Others | Stem | Spin | 1 | 0.004 | PLF311006 |
| *Laportea bulbifera* (Siebold & Zucc.) Wedd. | Hong huo ma | Urticaceae | Herb | Wild | Medicine, food | Tender leaf, root | Tender leaf: boiled or stewed with meat and eaten; root: decoction and orally taken, or medicinal bath, to treat edema, blister, traumatic injury, cough and cold, to relax tendons and activate collaterals, to dispel wind and eliminate dampness | 3 | 0.012 | LFE40406 |
| *Urtica fissa* E. Pritz. | Be huo ma | Urticaceae | Herb | Wild, spontaneous species | Medicine, food | Tender leaf, root | Tender leaf: boiled or stewed with meat and eaten; root: decoction and orally taken, or medicinal bath, to treat edema, blister, traumatic injury, cough and cold, to relax tendons and activate collaterals, to dispel wind and eliminate dampness | 4 | 0.016 | LFE30046 |
| *Glandularia* × *hybrida* (Groenland & Rümpler) G. L. Nesom & Pruski |  | Verbenaceae | Herb | Market | Ornamental | Flower | Ornamental | 3 | 0.012 | PLF408088 |
| *Verbena officinalis* L. | Tie ma bian | Verbenaceae | Herb | Spontaneous species | Medicine | Whole plant | Decoction and orally taken to treat cough and cold, gastroenteritis, gynecopathy, to clear heat and detoxify | 1 | 0.004 | LFE40174 |
| *Sambucus javanica* Reinw. ex Blume | Ba leng ma | Viburnaceae | Herb | Spontaneous species | Medicine | Whole plant | Mashed and external applied, or decoction or soaked in wine and orally taken, to dispel wind and eliminate dampness, to relax tendons and activate collaterals, to treat traumatic injury, tuberculosis | 7 | 0.029 | LFE40341 |
| *Viburnum plicatum* Thunb. | Xiu qiu hua | Viburnaceae | Shrub | Market, neighborhood | Ornamental | Flower | Ornamental | 9 | 0.037 | PLF404012 |
| *Viola philippica* Cav. | Hua kou jian | Violaceae | Herb | Spontaneous species | Medicine | Whole plant | Mashed and external applied for hemostasis, to treat detumescence, blister, centipede bite; medicinal bath to dispel wind and eliminate dampness | 1 | 0.004 | LFE40132 |
| *Vitis vinifera* L. | Pu tao | Vitaceae | Liana | Market | Food | Fruit | Eaten directly | 15 | 0.062 | PLF406010 |
| *Alpinia japonica* (Thunb.) Miq. | Xiao gan zi | Zingiberaceae | Herb | Wild, neighborhood | Medicine | Rhizome | Decoction and orally taken to treat cough and cold, dyspepsia, to dispel wind and eliminate dampness | 15 | 0.062 | LFE40073 |
| *Globba racemosa* Sm. | Ye yang huo | Zingiberaceae | Herb | Spontaneous species | Ornamental | Flower | Ornamental | 2 | 0.008 | PLF408050 |
| *Hedychium coronarium* J. König | Cao guo | Zingiberaceae | Herb | Self-preservation | Medicine, food, ornamental | Fruit, flower, rhizome | Fruit: as spice; flower: make soup and eaten; rhizome: stir-fried or stewed with meat and eaten to treat headache, soaked in wine and orally taken to treat aching lumbus; ornamental | 12 | 0.049 | LFE40285 |
| *Zingiber officinale* Roscoe | Jiang | Zingiberaceae | Herb | Self-preservation | Medicine, food, trade | Rhizome | Decoction and orally taken to treat cough and cold; as spice or as pickle; trade it at the market | 133 | 0.547 | PLF408014 |
| *Zingiber striolatum* Diels | Yang huo | Zingiberaceae | Herb | Neighborhood | Medicine, food, ornamental, trade | Flower, whole plant | Flower: stir-fried or made cold dishes and eaten; decoction and external applied to treat eczema; trade it at the market; whole plant: ornamental | 116 | 0.477 | LFE30023 |
